# Towards efficient verification of population protocols

**DOI:** 10.1007/s10703-021-00367-3

**Published:** 2021-04-14

**Authors:** Michael Blondin, Javier Esparza, Stefan Jaax, Philipp J. Meyer

**Affiliations:** 1 Département d’informatique, Université de Sherbrooke, 2500 boulevard de l’Université Sherbrooke, Québec, J1K 2R1 Canada; 2 Fakultät für Informatik, Technische Universität München, Boltzmannstraße 3, 85748 Garching bei München, Germany

**Keywords:** Population protocols, Automated verification, Termination

## Abstract

Population protocols are a well established model of computation by anonymous, identical finite-state agents. A protocol is well-specified if from every initial configuration, all fair executions of the protocol reach a common consensus. The central verification question for population protocols is the *well-specification problem*: deciding if a given protocol is well-specified. Esparza et al. have recently shown that this problem is decidable, but with very high complexity: it is at least as hard as the Petri net reachability problem, which is TOWER-hard, and for which only algorithms of non-primitive recursive complexity are currently known. In this paper we introduce the class ${ WS}^3$ of well-specified strongly-silent protocols and we prove that it is suitable for automatic verification. More precisely, we show that ${ WS}^3$ has the same computational power as general well-specified protocols, and captures standard protocols from the literature. Moreover, we show that the membership and correctness problems for ${ WS}^3$ reduce to solving boolean combinations of linear constraints over ${\mathbb {N}}$. This allowed us to develop the first software able to automatically prove correctness for *all* of the infinitely many possible inputs.

## Introduction

Population protocols [2, 3] are a model of distributed computation by many anonymous finite-state agents. They were initially introduced to model networks of passively mobile sensors [2, 3], but are now also used to describe chemical reaction networks (see e.g. [11, 29]).

In each computation step of a population protocol, a fixed number of agents are chosen nondeterministically, and their states are updated according to a joint transition function. Since agents are anonymous and identical, the global state of a protocol is completely determined by the number of agents at each local state, called a configuration. A protocol computes a boolean value *b* for a given initial configuration $C_0$ if in all fair executions starting at $C_0$, all agents eventually agree to *b* — so, intuitively, population protocols compute by reaching consensus under a certain fairness condition. A protocol is *well-specified* if it computes a value for each of its infinitely many initial configurations (also called *inputs*). A well-specified protocol *computes* a predicate, namely the function that assigns to each input the corresponding consensus value. In a famous series of papers, Angluin *et al.* [2, 3] have shown that well-specified protocols compute exactly the predicates definable in Presburger arithmetic [2–5].

In this paper we search for efficient algorithms for the *well-specification problem* (Is a given protocol well specified?) and the *correctness problem* (Given a protocol and a predicate, does the protocol compute the predicate?). These are questions about an infinite family of finite-state systems. Indeed, for every input the semantics of a protocol is a finite graph with the reachable configurations as nodes. Deciding if the protocol reaches consensus for a fixed input, and if so which one, only requires to inspect one of these graphs, and can be done automatically using a model checker. This approach has been followed in a number of papers [10, 12, 30, 33], but it only shows well-specification or correctness for some inputs. There has also been work in formalizing well-specification and correctness proofs in interactive theorem provers [15], but this approach is not automatic: a human prover must first come up with a proof for each particular protocol.

Recently, the second author, together with other co-authors, has shown that the well-specification and correctness problems are decidable [20]. In particular, there is an algorithm that decides if for all inputs the protocol stabilizes to a boolean value. The proof uses deep results of the theory of Petri nets, a model very close to population protocols. However, the same paper shows that the two problems are at least as hard as the reachability problem for Petri nets, a famously difficult problem: the reachability problem has a non-elementary lower bound [14], i.e. it generally requires a tower of exponentials of time and space. Existing algorithms for the reachability problem are notoriously difficult to implement, and they are considered impractical for nearly all applications.

For this reason, in this paper we search for a class of well-specified protocols satisfying the following four properties:

- *No loss of expressive power*: the class should compute all Presburger-definable predicates.
- *Natural*: the class should contain most protocols discussed in the literature.
- *Feasible membership problem*: deciding membership in the class should have reasonable complexity.
- *Feasible correctness problem*: given a protocol in the class and a predicate, deciding if the protocol computes the predicate should have reasonable complexity.

The class ${ WS}$ of all well-specified protocols obviously satisfies  (a) and  (b), but not (c) or (d). So we introduce a new class ${ WS}^3$, standing for *Well-Specified Strongly Silent* protocols. We show that ${ WS}^3$ still satisfies (a) and (b), and then prove two results:

- The membership problem for ${ WS}^3$ is in the complexity class DP (the class of languages *L* such that $L = L_1 \cap L_2$ for some languages $L_1 \in \textsf {NP}$ and $L_2 \in \textsf {coNP}$). This is a dramatic improvement with respect to the non-elementary lower bound for the membership problem for ${ WS}$.
- The correctness problem for ${ WS}^3$ (i.e., deciding if a protocol of ${ WS}^3$ computes a given predicate) is also is in DP, when the predicate is expressed as a formula in the quantifier-free fragment of Presburger arithmetic extended with remainder constraints. Notice that this fragment is as expressive as Presburger arithmetic itself.

The class ${ WS}^3$ is defined in two steps. Loosely speaking, a protocol is *silent* if communication between agents eventually ceases, i.e., if every fair execution eventually reaches a configuration whose only successor is the configuration itself. In the first step we introduce and analyze the class ${ WS}^2$ of well-specified silent protocols. It is easy to see that a protocol belongs to ${ WS}^2$ iff it satisfies two properties for every initial configuration $C_0$: (i) every configuration reachable from $C_0$ can reach a terminal configuration, and (ii) there is a Boolean value *b* such that all agents of all terminal configurations reachable from $C_0$ agree to *b*. We show that ${ WS}^2$ still satisfies (a) and (b), but neither (c) nor (d). In the second step we exploit the characterization of ${ WS}^2$ in terms of (i) and (ii), and define ${ WS}^3$ as the class of protocols satisfying stronger versions of (i) and (ii). Loosely speaking, the stronger properties require (i) and (ii) to hold not only for the configurations reachable from $C_0$, but for larger, carefully chosen sets of configurations.

Our proofs that the membership and correctness problems belong to DP reduces them to checking (un)satisfiability of two systems of boolean combinations of linear constraints over the natural numbers. This allows us to implement our decision procedure on top of the constraint solver Z3 [28], yielding the first software able to automatically prove well-specification and correctness for *all* inputs. We have tested our implementation on the families of protocols studied in [10, 12, 30, 33]. These papers prove correctness for some inputs of protocols with up to 9 states and 28 transitions. Our approach proves correctness for all inputs of protocols with up to 20 states in less than one second, and protocols with 70 states and 2500 transitions in less than one hour. In particular, we can automatically prove correctness for *all* inputs in less time than previous tools needed to check *one single large input*.

The paper is organized as follows. Section 2 contains basic definitions. Section 3 introduces an intermediate class ${ WS}^2$ of Well-Specified Silent protocols, and shows that its membership problem is still as hard as for ${ WS}$. Section 4 characterizes ${ WS}^2$ in terms of two properties, and introduces ${ WS}^3$ (Well-Specified Strongly Silent protocols) as the class of protocols satisfying two stronger properties. The section then shows that the two new properties can be tested in NP and coNP, respectively, which leads to our main result: the membership and correctness problems for ${ WS}^3$ are in DP. Section 5 proves that ${ WS}^3$-protocols compute all Presburger predicates. Section 6 reports on our experimental results, and Sect. 7 presents conclusions.

## Preliminaries

*Multisets.* A *multiset* over a finite set *E* is a mapping $M :E \rightarrow {\mathbb {N}}$. The set of all multisets over *E* is denoted ${\mathbb {N}}^E$. For every $e \in E$, *M*(*e*) denotes the number of occurrences of *e* in *M*, and we extend this to sets $E' \subseteq E$ by setting $M(E') {\mathop {=}\limits ^{\text {def}}}\sum _{e \in E'} C(e)$. We sometimes denote multisets using a set-like notation, e.g.  is the multiset *M* such that $M(f) = 1$, $M(g) = 2$ and $M(e) = 0$ for every $e \in E \setminus \{f, g\}$. The *support* of $M \in {\mathbb {N}}^E$ is $\llbracket M\rrbracket {\mathop {=}\limits ^{\text {def}}}\{e \in E : M(e) > 0\}$. The *size* of $M \in {\mathbb {N}}^E$ is $|M| {\mathop {=}\limits ^{\text {def}}}\sum _{e \in E} M(e)$. Addition and comparison are extended to multisets componentwise, i.e. $(M \mathbin {+}M')(e) {\mathop {=}\limits ^{\text {def}}}M(e) + M'(e)$ for every $e \in E$, and $M \le M' {\mathop {\iff }\limits ^{\text {def}}}M(e) \le M(e)$ for every $e \in E$. We define multiset difference as $(M \mathbin {\ominus }M')(e) {\mathop {=}\limits ^{\text {def}}}\max (M(e) - M'(e), 0)$ for every $e \in E$. The empty multiset is denoted $\mathbf {0}$, and for every $e \in E$ we write $\mathbf {e} {\mathop {=}\limits ^{\text {def}}}$.

*Population protocols.* A *population* *P* over a finite set *E* is a multiset $P \in {\mathbb {N}}^E$ such that $|P| \ge 2$. The set of all populations over *E* is denoted by $\text {Pop}(E)$. A *population protocol* is a tuple ${\mathcal {P}}= (Q, T, X, I, O)$ where

- *Q* is a non-empty finite set of *states*,
- $T \subseteq Q^2 \times Q^2$ is a set of *transitions* such that for every $(p, q) \in Q^2$ there exists at least a pair $(p', q') \in Q^2$ such that $(p, q, p', q') \in T$,
- *X* is a non-empty finite *input alphabet*,
- $I : X \rightarrow Q$ is the *input function* mapping input symbols to states,
- $O : Q \rightarrow \{0, 1\}$ is the *output function* mapping states to boolean values.

Following the convention of previous papers, we call the populations of $\text {Pop}(Q)$ *configurations*. Intuitively, a configuration *C* describes a collection of identical finite-state *agents* with *Q* as set of states, containing *C*(*q*) agents in state *q* for every $q \in Q$, and at least two agents in total.

Pairs of agents^1^ interact using transitions. For every $t = (p, q, p', q') \in T$, we write $(p, q) \mapsto (p', q')$ to denote *t*, and we define  and . For every configuration *C* and transition $t \in T$, we say that *t* is *enabled* at *C* if $C \ge {\text {pre}(t)}$. Note that by definition of *T*, every configuration enables at least one transition. A transition $t \in T$ enabled at *C* can *occur*, leading to the configuration $C \mathbin {\ominus }{\text {pre}(t)} + {\text {post}(t)}$. Intuitively, a pair of agents in states ${\text {pre}(t)}$ move to states ${\text {post}(t)}$. We write $C \xrightarrow {t} C'$ to denote that *t* is enabled at *C* and that its occurrence leads to $C'$. A transition $t \in T$ is *silent* if ${\text {pre}(t)} = {\text {post}(t)}$, i.e., if it cannot change the current configuration.

For every sequence of transitions $w = t_1 t_2 \cdots t_k$, we write $C \xrightarrow {w} C'$ if there exists a sequence of configurations $C_0, C_1, \ldots , C_k$ such that $C = C_0 \xrightarrow {t_1} C_1 \cdots \xrightarrow {t_k} C_k = C'$. We also write $C \xrightarrow {} C'$ if $C \xrightarrow {t} C'$ for some transition $t \in T$, and call $C \xrightarrow {} C'$ a *step*. We write $C \xrightarrow {*} C'$ if $C \xrightarrow {w} C'$ for some $w \in T^*$. We say that $C'$ is *reachable from* *C* if $C \xrightarrow {*} C'$. An *execution* is an infinite sequence of configurations $C_0 C_1 \cdots$ such that $C_i \xrightarrow {} C_{i+1}$ for every $i \in {\mathbb {N}}$. An execution $C_0 C_1 \cdots$ is *fair* if for every step $C \xrightarrow {} C'$, if $C_i = C$ for infinitely many indices $i \in {\mathbb {N}}$, then $C_j = C$ and $C_{j+1} = C'$ for infinitely many indices $j \in {\mathbb {N}}$. We say that a configuration *C* is

- *terminal* if $C \xrightarrow {*} C'$ implies $C = C'$, i.e., if every transition enabled at *C* is silent;
- a *consensus configuration* if $O(p) = O(q)$ for every $p, q \in \llbracket C\rrbracket$.

For every consensus configuration *C*, let *O*(*C*) denote the unique output of the states in $\llbracket C\rrbracket$. An execution $C_0 C_1 \cdots$ *stabilizes* to $b \in \{0, 1\}$ if there exists $n \in {\mathbb {N}}$ such that $C_i$ is a consensus configuration and $O(C_i) = b$ for every $i \ge n$.

*Predicates computable by population protocols.* Every *input* $\nu \in \text {Pop}(X)$ is mapped to the configuration $I(\nu ) \in \text {Pop}(Q)$ defined byA configuration *C* is said to be *initial* if $C = I(\nu )$ for some input $\nu$. A population protocol is *well-specified* if for every input $\nu$, there exists $b \in \{0, 1\}$ such that every fair execution of ${\mathcal {P}}$ starting at $I(\nu )$ stabilizes to *b*. We say that ${\mathcal {P}}$ *computes* a predicate $\Pi :\text {Pop}(X) \rightarrow \{0,1\}$ if for every input $\nu$, every fair execution of ${\mathcal {P}}$ starting at $I(\nu )$ stabilizes to $\Pi (\nu )$. It is readily seen that ${\mathcal {P}}$ computes a predicate if and only if it is well-specified.

### Example 1

We consider the majority protocol of [4] as a running example. Initially, agents of the protocol can be in either state *A* or *B*. The protocol computes whether there are at least as many agents in state *B* as there are in state *A*. The states and the input alphabet are $Q = \left\{ A, B, a, b\right\}$ and $X = \{A, B\}$ respectively. The input function is the identity function, and the output function is given by $O(B) = O(b) = 1$ and $O(A) = O(a) = 0$. The set of transitions *T* consists of:

$$\begin{aligned} t_{AB}&= (A, B) \mapsto (a, b) \\ t_{Ab}&= (A, b) \mapsto (A, a) \\ t_{Ba}&= (B, a) \mapsto (B, b) \\ t_{ba}&= (b, a) \mapsto (b, b) \end{aligned}$$

and of silent transitions for the remaining pairs of states. Transition $t_{AB}$ ensures that every fair execution eventually reaches a configuration *C* such that $C(A) = 0$ or $C(B) = 0$. If $C(A) = 0 =C(B)$, then there were initially equally many agents in *A* and *B*. Transition $t_{ba}$ then acts as tie breaker, resulting in a terminal configuration populated only by *b*. If, say, $C(A) > 0$ and $C(B) = 0$, then there were initially more *A*s than *B*s, and $t_{Ab}$ ensures that every fair execution eventually reaches a terminal configuration populated only by *A* and *a*.

## Well-specified silent protocols

Silent protocols^2^ were introduced in [17]. Loosely speaking, a protocol is silent if communication between agents eventually ceases, i.e. if every fair execution eventually stays in the same configuration forever. Observe that a well-specified protocol need not be silent: fair executions may keep alternating from a configuration to another as long as they are consensus configurations with the same output.

### Definition 1

An execution $C_0 C_1 \cdots$ of a protocol is *silent* if there exist $n \in {\mathbb {N}}$ and a configuration *C* such that $C_i = C$ for every $i \ge n$. A population protocol ${\mathcal {P}}$ is *silent* if every fair execution of ${\mathcal {P}}$ is silent, regardless of the starting configuration. ${\mathcal {P}}$ is a ${ WS}^2$-*protocol* if it is well-specified and silent. We let ${ WS}^2$ denote the set of all ${ WS}^2$-protocols.

### Example 2

As explained in Example 1, every fair execution of the majority protocol is silent. This implies that the protocol is silent. If, for example, we add a new state $b'$ where $O(b') = 1$, and transitions $(b, b) \mapsto (b', b'), (b', b') \mapsto (b, b)$, then the protocol is no longer silent since the execution where two agents alternate between states *b* and $b'$ is fair but not silent.

Being silent is a desirable property. While in arbitrary protocols it is difficult to determine if an execution has already stabilized, in silent protocols it is simple: one just checks if the current configuration only enables silent transitions. Even though it is was not observed explicitly, the protocols introduced in [2] to characterize the expressive power of population protocols belong to ${ WS}^2$. Therefore, ${ WS}^2$-protocols can compute the same predicates as general ones.

Unfortunately, the following theorem shows that the membership problem for ${ WS}^2$ is still as hard as the reachability problem for Petri nets. The proof is very similar to the one of [21, Theorem 10]. However, since it requires several modifications at different places, we present it in the appendix.

### Proposition 1

The reachability problem for Petri nets is reducible in polynomial time to the membership problem for ${ WS}^2$. In particular, membership for ${ WS}^2$ has non-elementary complexity.

To circumvent this high complexity, in the next section we introduce a subclass of ${ WS}^2$ with the same expressive power, but a membership problem of much lower complexity.

## A finer class of silent well-specified protocols: ${ WS}^3$

${ WS}^2$-protocols are exactly the protocols satisfying the two following properties:

- Termination: for every reachable configuration *C*, there exists a terminal configuration $C'$ such that $C \xrightarrow {*} C'$.
- Consensus: for every initial configuration *C*, there exists $b \in \{0, 1\}$ such that every terminal configuration $C'$ reachable from *C* is a consensus configuration with output *b*, i.e. $C \xrightarrow {*} C'$ implies $O(C') = b$.

### Proposition 2

A protocol belongs to ${ WS}^2$ if and only if it satisfies Termination and Consensus.

### Proof

We prove a stronger result:

- A protocol is silent if and only if it satisfies Termination.
- A silent protocol is well-specified if and only if it satisfies Consensus.

((a) $\Rightarrow$): Follows immediately from the definitions.

((a) $\Leftarrow$): Let $C_0$ be an arbitrary configuration, and let $\gamma = C_0 C_1 C_2 \cdots$ be a fair execution of the protocol. Let ${\mathcal {C}}_\bot$ be the set of terminal configurations reachable from $C_0$. Since Termination holds for every reachable configuration, and so in particular for all of $C_1, C_2, \ldots$, all configurations of $\gamma$ can reach some cofiguration of ${\mathcal {C}}_\bot$.

For every $C_i$, let $d(C_i)$ be the length of a shortest path from $C_i$ to some configuration of ${\mathcal {C}}_\bot$. We claim that for every $n \ge 0$, there are infinitely many indices *i* such that $d(C_i) \le n$. Since there are only finitely many configurations reachable from $C_0$, say *K*, we have $d(C_i) \le K$ for every index $i \ge 0$. So it suffices to show that if there are infinitely many indices *i* such that $d(C_i) \le n$, then there are infinitely many indices *j* such that $d(C_j) \le n-1$.

Let $i_1 \le i_2 \le i_3 \cdots$ be an infinite collection of indices such that $d(C_{i_j}) \le n$ for every $j \ge 1$. By definition of *d*, for every configuration $C_{i_j}$ there is a step $C_{i_j}\xrightarrow {}C_{i_j}'$ such that $d(C_{i_j}')=n-1$. By fairness, we have $C_{i_j}' = C_{i_j +1}$ for infinitely many $j \ge 1$, and the claim is proved. By this claim, there are infinitely many indices *i* such that $d(C_i) \le 0$, i.e., $C_i \in {\mathcal {C}}_\bot$. Let $i_0$ be one of them. Since ${\mathcal {C}}_\bot$ only contains terminal configurations, we have $C_i = C_{i_0}$ for every $i \ge i_0$, and so $\gamma$ converges to $C_\bot$.

((b) $\Rightarrow$) Let ${\mathcal {P}}$ be a silent and well-specified protocol. Let $C_0$ be an initial configuration of ${\mathcal {P}}$, and let $C_0 C_1 \cdots C_n$ be a finite prefix of an execution such that $C_n$ is terminal. The execution $C_0 C_1 \cdots (C_n)^\omega$ is fair. Since the protocol is well-specified, $C_n$ is a consensus configuration.

((b) $\Leftarrow$) Let ${\mathcal {P}}$ be a silent protocol satisfying Consensus. By silentness, every fair execution starting at an initial configuration *C* eventually reaches a terminal configuration. Since ${\mathcal {P}}$ satisfies Consensus, all these configurations are consensus configurations, and moreover they all agree to the same boolean value. $\square$

We introduce the new class ${ WS}^3$ as a refinement of ${ WS}^2$ obtained by strengthening Termination and Consensus into two new properties called LayeredTermination and StrongConsensus. These properties are presented in Sects. 4.3.2 and  4.2, where we also show that their associated decision problems belong to NP and coNP respectively.

Before doing so, let us introduce some useful notions. Let ${\mathcal {P}}= (Q, T, X, I, O)$ be a population protocol. For every $S \subseteq T$, ${\mathcal {P}}[S]$ denotes the *protocol induced by* *S*, i.e. ${\mathcal {P}}[S] {\mathop {=}\limits ^{\text {def}}}(Q, S \cup T', X, I, O)$ where $T' {\mathop {=}\limits ^{\text {def}}}\left\{ (p, q, p, q) : p, q \in Q\right\}$ is added to ensure that any two states can interact. Let $\xrightarrow {}_{S}$ denote the transition relation of ${\mathcal {P}}[S]$. An *ordered partition* of *T* is a tuple $(T_1, T_2, \ldots , T_n)$ of nonempty subsets of *T* such that $T = \bigcup _{i=1}^n T_i$ and $T_i \cap T_j = \emptyset$ for every $1 \le i < j \le n$.

### Layered termination

We replace Termination by a stronger property called LayeredTermination, and show that deciding LayeredTermination belongs to NP. The definition of LayeredTermination is inspired by the typical structure of protocols found in the literature. Such protocols are organized in layers such that transitions of higher layers cannot be enabled by executing transitions of lower layers. For these protocols, Termination can be proven by showing that every (fair or unfair) execution of a layer is silent.

#### Definition 2

A population protocol ${\mathcal {P}}= (Q, T, X, I, O)$ satisfies LayeredTermination if there is an ordered partition $(T_1, T_2, \ldots , T_n)$ of *T* such that for every $i \in [n]$:

- every (fair or unfair) execution of ${\mathcal {P}}[T_i]$ is silent; and
- every (fair or unfair) execution of ${\mathcal {P}}[T_i]$ starting at a terminal configuration of
  ${\mathcal {P}}[T_1 \cup \cdots \cup T_{i-1}]$ contains only terminal configurations of ${\mathcal {P}}[T_1 \cup \cdots \cup T_{i-1}]$.

(Observe that both (a) and (b) must hold for *all* executions of ${\mathcal {P}}[T_i]$, starting at *any* configuration, whether it is reachable from some initial configuration or not.)

In other words, condition (a) states that every execution contaning only transitions of $T_i$ eventually reaches a configuration in which all non-silent transitions of $T_i$ are disabled. Condition (b) states that if all the the non-silent transitions of $T_1 \cup \cdots \cup T_{i-1}$ become disabled, they cannot be re-enabled by executing transitions of $T_i$.

#### Example 3

The majority protocol satisfies LayeredTermination. Indeed, consider the ordered partition $(T_1, T_2)$, where

$$\begin{aligned} T_1&= \{(A, B) \mapsto (a,b), (A, b) \mapsto (A, a)\} \\ T_2&= \{(B, a) \mapsto (B, b), (b, a) \mapsto (b, b)\}. \end{aligned}$$

All executions of ${\mathcal {P}}[T_1]$ and ${\mathcal {P}}[T_2]$ are silent. For every terminal configuration *C* of ${\mathcal {P}}[T_1]$, we have $\llbracket C\rrbracket \subseteq \{A, a\}$ or $\llbracket C\rrbracket \subseteq \{B, a, b\}$. In the former case, no transition of $T_2$ is enabled; in the latter case, taking transitions of $T_2$ cannot enable $T_1$.

#### Proposition 3

LayeredTermination implies Termination.

#### Proof

Let ${\mathcal {P}}= (Q, T, X, I, O)$ be a population protocol satisfying LayeredTermination, and let *C* be an arbitrary configuration of ${\mathcal {P}}$. Let $(T_1, T_2, \ldots , T_n)$ be the ordered partition of *T* for LayeredTermination. By condition (a) of Definition 2, there exists a sequence $w_1 \in T_1^*$ such that $C \xrightarrow {w_1} C_1$, and $C_1$ is a terminal configuration of ${\mathcal {P}}[T_1]$. By the same reasoning, there exists a sequence $w_2 \in T_2^*$ such that $C_1 \xrightarrow {w_2} C_2$, and $C_2$ is a terminal configuration of ${\mathcal {P}}[T_2]$; further, by condition (b) of Definition 2, $C_2$ is also a terminal configuration of ${\mathcal {P}}[T_1 \cup T_2]$. Iterating this process we find $C_1 \xrightarrow {w_1 \ldots w_n} C_n$ such that $C_n$ is a terminal configuration of ${\mathcal {P}}[T_1 \cup \cdots \cup T_n] = {\mathcal {P}}$. $\square$

In the rest of this section, we prove that checking LayeredTermination is in NP. We do this by showing that conditions (a) and (b) of Definition 2 can be checked in polynomial time.

#### Checking condition (a) of Definition 2

We recall a basic notion of Petri net theory recast in the terminology of population protocols. Let ${\mathcal {P}}= (Q, T, X, I, O)$ be a population protocol. By definition, for every step $C \xrightarrow {t} C'$ and every state *q* we have $C'(q) = C(q) + {\text {post}(t)}(q) - {\text {pre}(t)}(q)$. This equality can be extended to sequences of transitions. Let $|w|_t$ denote the number of occurrences of transition *t* in a sequence *w*. If $C \xrightarrow {w} C'$, then we have

1 $$\begin{aligned} C'(q) = C(q) + \sum _{t \in T} |w|_t \cdot ({\text {post}(t)}(q) - {\text {pre}(t)}(q)) \quad \text{ for } \text{ every } q \in Q. \end{aligned}$$

Intuitively, this *flow equation* states that, for every state *q*, the number $C'(q)$ of agents in *q* after the execution of *w* is equal to the initial number *C*(*q*) of agents, plus the number $\sum _{t \in T} |w|_t \cdot {\text {post}(t)}(q)$ of agents that enter *q* during the execution, minus the number $\sum _{t \in T} |w|_t \cdot {\text {pre}(t)}(q)$ of agents that leave *q*. In particular, the final configuration reached after executing *w* only depends on how many times each transition occurs in *w*, and not on the order in which the transitions occur.

In the following lemma we use the flow equation to characterize the protocols ${\mathcal {P}}$ for which there exists a configuration $C_0$ such that some non-silent execution starts at $C_0$. The proof makes crucial use of the fact that for every sequence *w* of transitions there exists some configuration $C_0$ that enables *w*; indeed, since each transition takes at most two agents from a given state, it suffices to put $2 \cdot |w|$ agents in each state.

##### Lemma 1

Let ${\mathcal {P}}= (Q, T, X, I, O)$ be a population protocol and let $\textit{NS}\subseteq T$ be its set of non-silent transitions. ${\mathcal {P}}$ has a configuration $C_0$ and a non-silent execution $C_0 C_1 \ldots$ iff there is a non-zero vector $\mathbf {x} : \textit{NS}\rightarrow {\mathbb {N}}$ such that $\sum _{t \in \textit{NS}} \mathbf {x}(t) \cdot ({\text {post}(t)}(q) - {\text {pre}(t)}(q)) \ge 0$ for every $q \in Q$.

##### Proof

$\Rightarrow$) Let $C_0 C_1 C_2 \cdots$ be a non-silent execution of ${\mathcal {P}}$. Since executing a silent transition does not change the current configuration, we can assume that all the transitions occurring in the execution are non-silent. Since the total number of agents of a configuration is left unchanged by transitions, there exist indices $j < k$ such that $C_j = C_k$. So $C_j \xrightarrow {w} C_j$ for some non-empty sequence *w* of non-silent transitions. Instantiating the flow equation with $C {\mathop {=}\limits ^{\text {def}}}C_j$ and $C' {\mathop {=}\limits ^{\text {def}}}C_j$ we get $\sum _{t \in T} |w|_t \cdot ({\text {post}(t)}(q) - {\text {pre}(t)}(q)) = 0$ for every state *q*. Define $\mathbf {x}(t) {\mathop {=}\limits ^{\text {def}}}|w|_t$ for every non-silent transition *t*. Observe that $\mathbf {x}$ is not zero because *w* is non-empty.

$\Leftarrow$) Without loss of generality, we can assume $\mathbf {x}(q) \in {\mathbb {N}}$. (If this is not the case, we multiply $\mathbf {x}$ by a suitable coefficient.) Let $w \in \textit{NS}^*$ be any sequence of transitions such that $|w|_t = \mathbf {x}(t)$ for every $t \in \textit{NS}$. Choose a configuration $C_0$ such that $C_0 \xrightarrow {w} C$ for some configuration *C*. Observe that $C_0$ exists, for example it suffices to take $C_0(q) > 2 \cdot |w|$ for every state *q*. By the flow Eq. (1), we have $C \ge C_0$, and as $|C| = |C_0|$ also $C = C_0$. It follows that $C_0 \xrightarrow {w} C_0 \xrightarrow {w} C_0 \xrightarrow {w} \cdots$ is a non-silent execution of ${\mathcal {P}}$, and we are done. $\square$

Lemma 1 immediately leads to a polynomial algorithm to check condition (a) of Definition 2:

##### Proposition 4

Let ${\mathcal {P}}= (Q, T, X, I, O)$ be a population protocol. Deciding whether an ordered partition $(T_1, T_2, \ldots , T_n)$ of *T* satisfies condition (a) of Definition 2 can be done in polynomial time.

##### Proof

By Lemma 1, we can check condition (a) by considering the protocols ${\mathcal {P}}[T_1]$, ${\mathcal {P}}[T_2]$, ..., ${\mathcal {P}}[T_n]$, one after the other, and checking for each ${\mathcal {P}}[T_i]$ the (non) existence of a rational vector $\mathbf {x}_i$ with $\mathbf {x}_i(t) \ge 0$ for every $t \in \textit{NS}{} \cap T_i$ satisfying the linear constraints of the lemma. Since we can scale each component $\mathbf {x}_i(t)$ with the least common multiple of all denominators, such a vector exists iff a vector over the natural numbers satisfying the constraints exists. Since linear programming is in ¶, the result follows. $\square$

#### Checking condition (b) of Definition 2

We first rephrase condition (b) in a more convenient form. Let ${\mathcal {P}}= (Q, T, X, I, O)$ be a population protocol, and let $U \subseteq T$ be a set of transitions. A configuration $C \in \text {Pop}(Q)$ is *U*-*dead* if it only enables silent transitions of *U*; in other words, if $t \in U$ and $C \xrightarrow {t} C'$, then $C' = C$. We say that ${\mathcal {P}}$ is *U*-*dead from* $C_0 \in \text {Pop}(Q)$ if every configuration reachable from $C_0$ is *U*-dead, i.e. $C_0 \xrightarrow {*} C$ implies that *C* is *U*-dead. Finally, we say that ${\mathcal {P}}$ is *U*-*dead* if it is *U*-dead from every *U*-dead configuration $C_0 \in \text {Pop}(Q)$. So, loosely speaking, if a protocol ${\mathcal {P}}$ is *U*-dead, then for every configuration either ${\mathcal {P}}$ can immediately execute some non-silent transition of *U*, or it can never execute any non-silent transition of *U*.

##### Lemma 2

Let ${\mathcal {P}}= (Q, T, X, I, O)$ be a population protocol, and let $(T_1, \ldots , T_n)$ be an ordered partition of *T*. Further, let $U_0 = \emptyset$, and for every $1 \le i \le n$ let $U_i = T_1 \cup T_2 \cdots \cup T_{i}$. We have: ${\mathcal {P}}$ satisfies condition (b) of Definition 2 iff for every $i \in [n]$ the protocol ${\mathcal {P}}[U_i]$ is $U_{i-1}$-dead

##### Proof

Assume condition (b) holds for some $i \in [n]$, and let $C_0$ be a $U_{i-1}$-dead configuration of ${\mathcal {P}}[U_i]$. By (b) no configuration reachable from $C_0$ by executing transitions of $T_i$ enables any transition of $U_{i-1}$, and so ${\mathcal {P}}[U_i]$ is $U_{i-1}$-dead from $C_0$. Conversely, assume ${\mathcal {P}}[U_i]$ is $U_{i-1}$-dead, and let $C_0$ be a terminal configuration of ${\mathcal {P}}[U_{i-1}]$. Then $C_0$ disables all non-silent transitions of $U_{i-1}$. Since ${\mathcal {P}}[U_i]$ is $U_{i-1}$-dead, no configuration reachable from $C_0$ by executing transitions of $T_i$ enables any non-silent transition of $U_{i-1}$. So every execution starting at $C_0$ contains only terminal configurations of ${\mathcal {P}}[U_{i-1}]$. $\square$

By this lemma, checking condition (b) in polynomial time reduces to giving a polynomial-time algorithm to check, given a protocol ${\mathcal {P}}= (Q, T, X, I, O)$ and a set $U \subseteq T$ of transitions, whether ${\mathcal {P}}$ is *U*-dead. (Indeed, in order to check (b) for every $i \in [n]$ it suffices to instantiate the algorithm with the protocols ${\mathcal {P}}[U_1], \ldots , {\mathcal {P}}[U_n]$ and the sets $U_0, \ldots , U_{n-1}$, respectively.) To this end, we first characterize the pairs ${\mathcal {P}}, U$ such that ${\mathcal {P}}$ is *U*-dead.

##### Lemma 3

Let ${\mathcal {P}}= (Q, T, X, I, O)$ be a protocol and let $U \subseteq T$ be a set of transitions. ${\mathcal {P}}$ is *U*-dead iff for every transition $s \in T \setminus U$ and every non-silent transition $u \in U$:

2 $$\begin{aligned} {\text {pre}(u')} \le {\text {pre}(s)} + ({\text {pre}(u)} \mathbin {\ominus }{\text {post}(s)}) \quad \text { for some non-silent transition } u' \in U . \end{aligned}$$

##### Proof

We prove that ${\mathcal {P}}$ is *not* *U*-dead iff there exists a transition $s \in T \setminus U$ and a non-silent transition $u \in U$ such that:

3 $$\begin{aligned} {\text {pre}(u')} \nleq {\text {pre}(s)} + ({\text {pre}(u)} \mathbin {\ominus }{\text {post}(s)}) \quad \text { for every non-silent transition } u' \in U . \end{aligned}$$

$\Leftarrow$) Suppose there exist $s \in T \setminus U$ and non-silent $u \in U$ such that (3) holds. Let $C_0 \in \text {Pop}(Q)$ be the configuration $C_0 {\mathop {=}\limits ^{\text {def}}}{\text {pre}(s)} + ({\text {pre}(u)} \mathbin {\ominus }{\text {post}(s)})$. By (3), $C_0$ does not enable any non-silent transition of *U*, and so $C_0$ is *U*-dead. Since $C_0 \ge {\text {pre}(s)}$, we have $C_0 \xrightarrow {s} C$ for $C = ({\text {pre}(u)} \mathbin {\ominus }{\text {post}(s)}) + {\text {post}(s)}$. Further, since ${\text {pre}(u)} \le C$, the configuration *C* enables *u*. So $C_0$ is not *U*-dead, and therefore ${\mathcal {P}}$ is not *U*-dead.

$\Rightarrow$) Assume ${\mathcal {P}}$ is not *U*-dead. Then there exist steps $C_0 \xrightarrow {s_1} C_1 \xrightarrow {s_2} \cdots \xrightarrow {s_n} C_n$ such that $C_0, \ldots , C_{n-1}$ are *U*-dead, $s_1, s_2, \dots , s_n \in T \setminus U$, and $C_n$ is not *U*-dead. Let $u \in U$ be any non-silent transition enabled at $C_n$, i.e. such that ${\text {pre}(u)} \le C_n$. We prove by contradiction that (3) holds for $s:= s_n$ and this transition *u*. Suppose there exists some non-silent $u' \in U$ such that ${\text {pre}(u')} \le {\text {pre}(s_n)} + ({\text {pre}(u)} \mathbin {\ominus }{\text {post}(s_n)})$. We have

$$\begin{aligned} {\text {pre}(u')}&\le {\text {pre}(s_n)} + ({\text {pre}(u)} \mathbin {\ominus }{\text {post}(s_n)}) \\&\le {\text {pre}(s_n)} + (C_n \mathbin {\ominus }{\text {post}(s_n)})&\text {(by } {\text {pre}(u)} \le C_n) \\&\le C_n \mathbin {\ominus }{\text {post}(s_n)} + {\text {pre}(s_n)} \\&= C_{n-1}&\text {(by }C_{n-1} \xrightarrow {s_n} C_n)\ . \end{aligned}$$

Therefore, $C_{n-1} \xrightarrow {u'} C$ for some configuration *C*. Moreover, $C \ne C_{n-1}$ because $u'$ is non-silent. This contradicts the fact that $C_{n-1}$ is *U*-dead, hence (3) holds. $\square$

##### Proposition 5

Let ${\mathcal {P}}= (Q, T, X, I, O)$ be a population protocol. Deciding whether an ordered partition $(T_1, \ldots , T_n)$ of *T* satisfies condition (b) of Definition 2 can be done in polynomial time.

##### Proof

Let $U_0 = \emptyset$, and for every $1 \le i \le n$ let $U_i = T_1 \cup T_2 \cdots \cup T_{i}$. By Lemma 2, condition (b) holds for a given $i \in [n]$ iff the protocol ${\mathcal {P}}[U_i]$ is $U_{i-1}$-dead. By Lemma 3, ${\mathcal {P}}[U_i]$ is $U_{i-1}$-dead iff the condition of the lemma holds for ${\mathcal {P}}:= {\mathcal {P}}[U_i]$ and $U:=U_{i-1}$, in other words, if (2) holds for every pair $(s, u) \in T_i \times U_{i-1}$ of transitions. Since (2) can be checked in polynomial time, and the number of pairs is also polynomial, the result follows. $\square$

Propositions 4 and 5 yield an NP procedure to decide LayeredTermination. Indeed, it suffices to guess an ordered partition and to check whether it satisfies conditions (a) and (b) of Definition 2 in polynomial time.

##### Corollary 1

Deciding if a protocol satisfies LayeredTermination is in NP.

Via a straightforward reduction from 3-SAT (satisfiability of a formula in conjunctive normal form, with 3 literals per clause), we show in in Appendix 8.2 that deciding LayeredTermination is NP-hard. Thus we obtain:

##### Proposition 6

Deciding if a protocol satisfies LayeredTermination is NP-complete.

### Strong consensus

To overcome the high complexity of reachability in population protocols, we strengthen Consensus by replacing the reachability relation in its definition by an *overapproximation*, i.e., a relation  over configurations such that $C \xrightarrow {*} C'$ implies . Observe that the flow equations provide an over-approximation of the reachability relation. Indeed, as mentioned earlier, if $C \xrightarrow {*} C'$, then there exists $\mathbf {x} : T \rightarrow {\mathbb {N}}$ such that $(C, C', \mathbf {x})$ satisfies all of the flow equations. However, this over-approximation alone is too crude for the verification of protocols.

#### Example 4

Consider the configurations $$C = $$ and $$C' = $$ of the majority protocol. The flow equations are satisfied by the mapping $$\mathbf {x}$$ such that $$\mathbf {x}(t_{AB}) = \mathbf {x}(t_{Ab}) = 1$$ and $$\mathbf {x}(t_{Ba}) = \mathbf {x}(t_{ba}) = 0$$. Yet, $$C \xrightarrow {*} C'$$ does not hold.

To obtain a finer reachability over-approximation, we introduce so-called traps and siphons constraints borrowed from the theory of Petri nets [16, 18, 19]. These constraints have been successfully applied to a number of analysis problems (see e.g. [6, 18, 19]). Intuitively, for some subset of transitions $U \subseteq T$, a *U*-trap is a set of states $P \subseteq Q$ such that every transition of *U* that removes an agent from *P* also moves an agent into *P*. Conversely, a *U*-siphon is a set $P \subseteq Q$ such that every transition of *U* that moves an agent into *P* also removes an agent from *P*. More formally, for every $R \subseteq Q$, let ${^\bullet R} {\mathop {=}\limits ^{\text {def}}}\{t \in T : \llbracket {\text {post}(t)}\rrbracket \cap R \not = \emptyset \}$ and ${R^\bullet } {\mathop {=}\limits ^{\text {def}}}\{t \in T : \llbracket {\text {pre}(t)}\rrbracket \cap R \not = \emptyset \}$. *U*-siphons and *U*-traps are defined as follows:

#### Definition 3

A subset of states $P \subseteq Q$ is a *U*-*trap* if ${P^\bullet } \cap U \subseteq {^\bullet P}$, and a *U*-*siphon* if ${^\bullet P} \cap U \subseteq {P^\bullet }$.

For every configuration $C \in \text {Pop}(Q)$ and $P \subseteq Q$, let $C(P) {\mathop {=}\limits ^{\text {def}}}\sum _{q \in P} C(q)$. Consider a sequence of steps $C_0 \xrightarrow {t_1} C_1 \xrightarrow {t_2} \cdots \xrightarrow {t_n} C_n$ where $t_1, \ldots , t_n \in U$. It follows from Definition 3 that if some transition $t_i$ moves an agent to a *U*-trap *P*, then $C_j(P) > 0$ for every $j \ge i$. Similarly, if some transition $t_i$ removes an agent from a *U*-siphon, then $C_j(P) > 0$ for every $j < i$. In particular:

#### Observation 1

Let $U \subseteq T$, let *C* and $C'$ be configurations, and let *w* be a sequence such that $C \xrightarrow {w} C'$ and $|w|_t > 0$ for every $t \in U$. For every *U*-trap *P*, if $C'(P) = 0$, then ${^\bullet P} \cap U = \emptyset$. For every *U*-siphon *P*, if $C(P) = 0$, then ${P^\bullet } \cap U = \emptyset$.

We obtain a necessary condition for $C \xrightarrow {*} C'$ to hold, which we call potential reachability:

#### Definition 4

Let $C, C'$ be two configurations, let $\mathbf {x} : T \rightarrow {\mathbb {N}}$, and let $U = \llbracket \mathbf {x}\rrbracket$. We say that $C'$ is *potentially reachable from* *C* *through* $\mathbf {x}$, denoted , if

- the flow equation $C'(q) = C(q) + \sum _{t \in T} \mathbf {x}(t) \cdot ({\text {post}(t)}(q) - {\text {pre}(t)}(q))$ holds for every state $q \in Q$,
- $C'(P) = 0$ implies ${^\bullet P} \cap U = \emptyset$ for every *U*-trap *P*, and
- $C(P) = 0$ implies ${P^\bullet } \cap U = \emptyset$ for every *U*-siphon *P*.

#### Example 5

Let us reconsider Example 4. Let $U = \llbracket \mathbf {x}\rrbracket = \{t_{AB}, t_{Ab}\}$ and $P = \{A, b\}$. Recall that $t_{AB} = (A, B) \mapsto (a, b)$ and $t_{Ab} = (A, b) \mapsto (A, a)$. We have ${P^\bullet } \cap U = U$ which implies that *P* is a *U*-trap. This means that Definition 4(b) is violated as $C'(P) = 0$ and ${^\bullet P} \cap U = U \not = \emptyset$. Therefore,  does not hold.

We write  if  for some $\mathbf {x} : T \rightarrow {\mathbb {N}}$. As an immediate consequence of Observation 1, for every configurations *C* and $C'$, if $C \xrightarrow {*} C'$, then . This allows us to strengthen Consensus by redefining it in terms of potential reachability instead of reachability:

#### Definition 5

A protocol satisfies StrongConsensus if for every initial configuration *C*, there exists $b \in \{0, 1\}$ such that every terminal configuration $C'$ potentially reachable from *C* is a consensus configuration with output *b*, i.e.  implies $O(C') = b$.

Since the number of *U*-traps and *U*-siphons of a protocol can be exponential in the number of states, checking trap and siphon constraints by enumerating them may take exponential time. Fortunately, this can be avoided. By definition, it follows that the union of two *U*-traps is again a *U*-trap, and similarly for siphons. Therefore, given a configuration *C*, there exists a unique maximal *U*-siphon $P_\text {max}$ such that $C(P_\text {max}) = 0$, and a unique maximal *U*-trap $P'_\text {max}$ such that $C(P'_\text {max}) = 0$. Moreover, $P_\text {max}$ and $P'_\text {max}$ can be computed in linear time by means of a simple greedy algorithm (see e.g. [16, Ex. 4.5]). This simplifies the task of checking traps and siphons constraints, and yields a coNP procedure for testing StrongConsensus:

#### Proposition 7

Deciding if a protocol satisfies StrongConsensus is in coNP.

#### Proof

Testing whether a protocol *does not* satisfy StrongConsensus can be done by guessing $C_0, C, C' \in \text {Pop}(Q)$, $q, q' \in Q$ and $\mathbf {x}, \mathbf {x}' : T \rightarrow {\mathbb {N}}$, and testing whether

- $C_0$ is initial, *C* is terminal, $C'$ is terminal, $q \in \llbracket C\rrbracket$, $q' \in \llbracket C'\rrbracket$, $O(q) \not = O(q')$, and
and .

Since there is no a priori bound on the size of $C_0, C, C'$ and $\mathbf {x}, \mathbf {x}'$, we guess them carefully. First, we guess whether $D(p) = 0$, $D(p) = 1$ or $D(p) \ge 2$ for every $D \in \{C_0, C, C'\}$ and $p \in Q$. This gives enough information to test (a). Then, we guess $\llbracket \mathbf {x}\rrbracket$ and $\llbracket \mathbf {x}'\rrbracket$. This allows to test traps/siphons constraints as follows. Let $U {\mathop {=}\limits ^{\text {def}}}\llbracket \mathbf {x}\rrbracket$, let $P_\text {max}$ be the maximal *U*-trap such that $C(P_\text {max}) = 0$, and let $P'_\text {max}$ be the maximal *U*-siphon such that $C_0(P'_\text {max}) = 0$. Conditions (b) and (c) of Definition 4 hold if and only if ${^\bullet (P_\text {max})} \cap U = \emptyset$ and ${(P'_\text {max})^\bullet } \cap U = \emptyset$, which can be tested in polynomial time. The same is done for $\mathbf {x}'$. If (a) and siphons/traps constraints hold, we build the system ${\mathcal {S}}$ of linear equations/inequalities obtained from the conjunction of the flow equations together with the constraints already guessed. By standard results on integer linear programming (see e.g. [32, Sect. 17]), if ${\mathcal {S}}$ has a solution, then it has one of polynomial size, and hence we may guess it. $\square$

### ${ WS}^3$-protocols

We introduce the class ${ WS}^3$ of protocols:

#### Definition 6

A protocol belongs to ${ WS}^3$ if it satisfies LayeredTermination and StrongConsensus.

Since ${ WS}^3$ $\subseteq$ ${ WS}^2$ $\subseteq$ ${ WS}$ holds, every ${ WS}^3$-protocol is well-specified. We study the computational complexity of the membership problem and correctness problems for ${ WS}^3$:

- **Membership**: Given a protocol, does it belong to ${ WS}^3$?
- **Correctness**: Given a protocol and a predicate, does the protocol belong to ${ WS}^3$ and compute the predicate?

We first show that the membership problem belongs to the class DP. Recall that a language *L* belongs to DP if there exist languages $L_1 \in \textsf {NP}$ and $L_2 \in \textsf {coNP}$ such that $L = L_1 \cap L_2$ [31].

#### Theorem 2

The membership problem for ${ WS}^3$-protocols is in DP.

#### Proof

Let $L_1$ and $L_2$ be the languages of population protocols satisfying LayeredTermination and StrongConsensus, respectively. By Corollary 1 and Proposition 7, we have ${ WS}^3 = L_1 \cap L_2$ where $L_1 \in \textsf {NP}$ and $L_2 \in \textsf {coNP}$, and we are done. $\square$

Let us now consider the correctness problem. Recall that a protocol over an input alphabet *X* computes a predicate $\text {Pop}(X) \rightarrow \{0,1\}$. As mentioned in the introduction, Angluin *et al.* [4] have shown that for every finite input alphabet *X*, a predicate $\text {Pop}(X) \rightarrow \{0,1\}$ is computable by a population protocol over *X* if and only if it is definable in Presburger arithmetic, the first-order theory of addition [2, 4].

#### Definition 7

A *threshold constraint* over a set of variables *X* is an expression of the form $\sum _{i=1}^k a_i x_i < c$, where $a_1, \ldots , a_k, c$ are integers represented in binary, and $x_1, \ldots , x_k \in X$. A *Presburger formula* over *X* is an expression $\varphi$ over the syntax

$$\begin{aligned} \varphi {:}{:}{=} t \mid \lnot \varphi _1 \mid \varphi _1 \wedge \varphi _2 \mid \varphi _1 \vee \varphi _2 \mid \exists x \, \varphi _1 \mid \forall x \, \varphi _1 \end{aligned}$$

where *t* is a threshold constraint over *X*, and $x \in X$.

A Presburger formula $\varphi (x_1, \ldots , x_n)$ with free variables $x_1, \ldots x_n$ is interpreted over $\text {Pop}(\{x_1, \ldots , x_n\})$, i.e., over the mappings^3^$\{x_1, \ldots , x_n\} \rightarrow {\mathbb {N}}$. Given $\nu \in \text {Pop}(\{x_1, \ldots , x_n\})$, the satisfaction relation $\nu \models \varphi (x_1, \ldots , x_n)$ is inductively defined as usual; in particular, $\nu ~\models ~\sum _{i=1}^n a_i x_i < c$ iff $\sum _{i=1}^n a_i \nu (x_i) < c$. We let $\llbracket \varphi \rrbracket$ denote the predicate ${\mathbb {N}}^n \rightarrow \{0,1\}$ given by $\llbracket \varphi \rrbracket (\nu ) =1$ iff $\nu \models \varphi$. Given a finite alphabet *X*, a predicate $\Pi :\text {Pop}(X) \rightarrow \{0,1\}$ is a *Presburger predicate* if $\Pi =\llbracket \varphi \rrbracket$ for some Presburger formula $\varphi$ with *X* as set of free variables. Two Presburger formulas $\varphi ,\psi$ are *equivalent* if $\llbracket \varphi \rrbracket = \llbracket \psi \rrbracket$.

In the rest of the section we study the problem of whether a given protocol ${\mathcal {P}}$ is in ${ WS}^3$ and computes a Presburger predicate specified by a Presburger formula $\varphi$.

By definition, the protocols of ${ WS}^3$ are those satisfying LayeredTermination and StrongConsensus. Given a Presburger formula $\varphi$ over a set *X* of variables, we characterize the protocols of ${ WS}^3$ that compute the predicate $\llbracket \varphi \rrbracket$. For this we introduce a new property of a protocol ${\mathcal {P}}=(Q,T,X,I, O)$, similar to StrongConsensus:

#### Definition 8

A protocol satisfies Strong-$\varphi$-Consensus if for for every input $\nu \in \text {Pop}(X)$, every terminal configuration potentially reachable from $I(\nu )$ is a consensus configuration with output $\llbracket \varphi \rrbracket (\nu )$.

#### Proposition 8

Let $\varphi$ be a Presburger formula. A protocol ${\mathcal {P}}$ is in ${ WS}^3$ and computes $\llbracket \varphi \rrbracket$ iff ${\mathcal {P}}$ satisfies LayeredTermination and Strong-$\varphi$-Consensus.

#### Proof

$\Rightarrow$) Assume ${\mathcal {P}}$ is in ${ WS}^3$ and computes $\llbracket \varphi \rrbracket$. Fix some input $\nu \in \text {Pop}(X)$. Since ${\mathcal {P}}$ is in ${ WS}^3$, it satisfies LayeredTermination and StrongConsensus. By LayeredTermination and Proposition 3 we have that some terminal configuration $C_\bot$ is reachable from $I(\nu )$. Since ${\mathcal {P}}$ computes $\llbracket \varphi \rrbracket$, it must hold that $O(C_\bot ) = \llbracket \varphi \rrbracket (\nu )$. Potential reachability is an over-approximation of reachability, hence reachability of $C_\bot$ implies potential reachability of $C_\bot$ from $I(\nu )$. By StrongConsensus, all potentially reachable terminal configurations are in the same consensus as $C_\bot$. So all potentially reachable terminal configurations form the consensus $O(C_\bot ) = \llbracket \varphi \rrbracket (\nu )$ and Strong-$\varphi$-Consensus follows.

$\Leftarrow$) If ${\mathcal {P}}$ satisfies Strong-$\varphi$-Consensus, then it also ${\mathcal {P}}$ satisfies StrongConsensus, as Strong-$\varphi$-Consensus is a specialization of StrongConsensus. So ${\mathcal {P}}$ belongs to ${ WS}^3$. Further, as ${\mathcal {P}}$ satisfies LayeredTermination, for every input $\nu \in \text {Pop}(X)$, every fair execution of ${\mathcal {P}}$ starting at $I(\nu )$ reaches a terminal configuration. Since ${\mathcal {P}}$ satisfies ${\textsc {Strong}}-\varphi -{\textsc {Consensus}}$, every fair execution starting at $I(\nu )$ stabilizes to $\llbracket \varphi \rrbracket (\nu )$. So ${\mathcal {P}}$ computes $\llbracket \varphi \rrbracket$. $\square$

#### Complexity of the correctness problem

The complexity of the correctness problem for ${ WS}^3$ depends on the formalism used to represent Presburger predicates. We choose to represent them as boolean combinations of threshold and remainder constraints. Before explaining why, we introduce some definitions.

##### Definition 9

A *remainder constraint* over a set of variables *X* is an expression of the form $\sum _{i=1}^k a_i x_i \equiv c \pmod {m}$, where $a_1, \ldots , a_k, c, m$ are integers represented in binary with $0 \le c < m$ and $m \ge 2$, and $x_1, \ldots , x_k \in X$. A *TR-constraint* is a boolean combination of threshold and remainder constraints.

There are two other formalisms with the same expressive power as Presburger formulas, i.e., able to express exactly the Presburger predicates: TR-constraints and semilinear sets. Indeed, by the quantifier-elimination procedure for Presburger arithmetic, every Presburger formula is equivalent to a TR-constraint [13]^4^. Further, the set of solutions of a Presburger formula is semilinear, and so it can be finitely represented by listing the roots and periods of the linear sets that compose it [23].

We choose TR-constraints as specification formalism, because it provides the best trade-off between readability and tool support. Semilinear sets are difficult to parse by humans. Full Presburger arithmetic is very succinct, but it has two problems: from the theoretical point of view, the complexity of the correctness problem is dominated by the complexity of the satisfiability problem for Presburger arithmetic, which lies between 2-NEXP and 2-EXPSPACE, and is thus very high [7, 22]; from the practical point of view, constraint solvers for Presburger arithmetic are much less efficient than those for TR-constraints. Moreover, the standard predicates studied in the literature are already naturally expressed with TR-constraints. For all these reasons, in the rest of the paper we specify a predicate as a TR-constraint $\varphi (X)$ with *X* as set of free variables.

We wish to prove that deciding if ${\mathcal {P}}$ satisfies Strong-$\varphi$-Consensus, where $\varphi$ is a TR-constraint, is in coNP. For this we need a lemma.

##### Lemma 4

The satisfiability problem for TR-constraints is in NP.

##### Proof

Let $\varphi (x_1, \ldots , x_n)$ be a TR-constraint. We show that $\varphi (x_1, \ldots , x_n)$ is equivalent to an existential Presburger formula of length $O(|\varphi |)$, and use that the satisfiability problem for existential Presburger arithmetic is NP-complete [24].

By pushing negations inside if necessary, we can transform $\varphi$ into a TR-constraint where negations only appear in front of threshold or remainder constraints. We have that

$$\begin{aligned} \sum _{i=1}^k a_i \cdot x_i \equiv c \pmod {m}&\text { if{}f } \exists y : \left( m \cdot y + c = \sum _{i=1}^k a_i \cdot x_i \right) ,\text { and }\\ \sum _{i=1}^k a_i \cdot x_i \not \equiv c \pmod {m}&\text { if{}f } \exists y,z : \left( m \cdot y + z = \sum _{i=1}^k a_i \cdot x_i \right) \wedge \left( 0 \le z < m \right) \wedge \left( z \ne c \right) . \end{aligned}$$

It is easy to see that $\le$, $=$ and $\ne$ can be expressed as boolean combinations of threshold constraints using <. Since existential quantifiers can be moved to the front of the formula, we are done. $\square$

##### Proposition 9

Let $\varphi (X)$ be a TR-constraint, and let ${\mathcal {P}}=(Q,T,X,I, O)$ be a population protocol. Deciding if ${\mathcal {P}}$ satisfies Strong-$\varphi$-Consensus is in coNP.

##### Proof

Let $\Pi = \llbracket \varphi \rrbracket .$ Testing whether a protocol *does not* satisfy Strong-$\varphi$-Consensus can be done by guessing $\mathbf {x} \in \text {Pop}(X)$, $C \in \text {Pop}(Q)$, $\mathbf {z} :T \rightarrow {\mathbb {N}}$, and testing whether

- *C* is terminal,
,
- $$O(C) \ne \Pi (\mathbf {x})$$.

Since there is no a priori bound on the size of $\mathbf {x}$, *C* and $\mathbf {z}$, we guess them in an analogous manner to the proof of Proposition 7. First, we guess whether $C(p) = 0$, $C(p) = 1$ or $C(p) \ge 2$ for every $p \in Q$. This gives enough information to test (a). Then, we guess $\llbracket \mathbf {x}\rrbracket$ and $\llbracket \mathbf {z}\rrbracket$. This allows to test traps/siphons constraints in the same way as in the proof of Proposition 7. If siphons/traps constraints hold, we build the system ${\mathcal {S}}$ of linear equations/inequalities obtained from the conjunction of the flow equations together with the constraints already guessed. For (c) we distinguish the two cases: $O(C) = 0$ or $O(C) = 1$. The disjunction of the two cases along with the constraints ${\mathcal {S}}$ yields

$$\begin{aligned} \psi {\mathop {=}\limits ^{\text {def}}}{\mathcal {S}}(C, \mathbf {x}) \wedge \left( (O(C) = 0 \wedge \varphi (\mathbf {x})) \vee (O(C) = 1 \wedge \lnot \varphi (\mathbf {x})) \right) . \end{aligned}$$

Since $\varphi$ is a TR-constraint, so is $\psi$. By Lemma 4, satisfiability of $\psi$ can be decided in non-deterministic polynomial time. From this, and the fact that Strong-$\varphi$-Consensus holds precisely if $\psi$ is unsatisfiable for all guesses of $\mathbf {x}$, *C* and $\mathbf {z}$, we obtain that Strong-$\varphi$-Consensus is in coNP. $\square$

##### Corollary 2

Let $\varphi (\Sigma )$ be a TR-constraint and let ${\mathcal {P}}$ be a protocol. Deciding if ${\mathcal {P}}$ is in ${ WS}^3$ and computes $\llbracket \varphi \rrbracket$ is in DP.

##### Proof

Deciding whether ${\mathcal {P}}$ is in ${ WS}^3$ and computes $\llbracket \varphi \rrbracket$ is by Proposition  8 equivalent to deciding whether ${\mathcal {P}}$ satisfies LayeredTermination and Strong-$\varphi$-Consensus. Let $L_1$ and $L_2$ be the languages of population protocols and formulas satisfying LayeredTermination and Strong-$\varphi$-Consensus, respectively. By Corollary 1 and Proposition 9 we have that $L_1 \in \textsf {NP}$ and $L_2 \in \textsf {coNP}{}$, and thus $L_1 \cap L_2 \in \textsf {DP}{}$, and we are done. $\square$

#### Determining the predicate computed by a ${ WS}^3$ protocol

We show that the procedures for checking LayeredTermination and StrongConsensus shown in Sect. 4.2, respectively, not only determine whether a given protocol belongs to ${ WS}^3$; when the protocol does belong to ${ WS}^3$, we can also use them to extract a Presburger formula for the predicate computed by the protocol. We first prove:

##### Proposition 10

Let ${\mathcal {P}}= (Q, T, X, I, O)$ be a protocol in ${ WS}^3$, and let $\Pi$ be the predicate computed by ${\mathcal {P}}$. For every input $\nu \in \text {Pop}(X)$, we have $\Pi (\nu ) = 1$ iff there exists a terminal configuration *C* such that $O(C)=1$ and .

##### Proof

Fix an arbitrary input $\nu$.

$\Rightarrow$) Assume $\Pi (\nu ) = 1$. By definition, every fair execution starting at $I(\nu )$ eventually stabilizes to 1. Since ${\mathcal {P}}$ belongs to ${ WS}^3$, it is silent, and so every fair execution eventually reaches a terminal configuration with output 1. So $I(\nu ) \xrightarrow {*} C$ for some configuration *C* such that $O(C)=1$. Since reachability implies potential reachability,  holds as well.

$\Leftarrow$) Assume there exists a terminal configuration *C* such that $O(C)=1$ and . We show that every fair execution $I(\nu ) \, C_1 \, C_2 \ldots$ of ${\mathcal {P}}$ stabilizes to 1. Since ${\mathcal {P}}$ satisfies LayeredTermination it also satisfies Termination, and therefore the execution eventually reaches some terminal configuration $C_i$. In particular, we have $I(\nu ) \xrightarrow {*} C_i$, which implies . Since ${\mathcal {P}}$ satisfies StrongConsensus, and both *C* and $C_i$ are potentially reachable terminal configurations, we have $O(C_i)=O(C)=1$. So the execution stabilizes to 1. $\square$

Given a protocol ${\mathcal {P}}$, it is easy to give Presburger formulas $\textit{Term}(C)$ and $\textit{Output1}(C)$ that hold iff *C* is a terminal configuration and a configuration with output 1, respectively. Moreover, it follows from the proof of Proposition 7 that there exists a Presburger formula $\textit{PotReach}(C, C')$ that holds iff . By Proposition 10, the formula

$$\begin{aligned} \varphi (\nu ) = \exists C :\textit{PotReach}(I(\nu ), C) \wedge \textit{Term}(C) \wedge \textit{Output1}(C) \end{aligned}$$

characterizes the protocol computed by ${\mathcal {P}}$.

## ${ WS}^3$ is as expressive as ${ WS}$

Recall that Angluin *et al.* have shown that a predicate is computable by a population protocol if and only if it is definable in Presburger arithmetic [2, 4]. In particular, [2] shows how to construct a protocol for a given Presburger-definable predicate. The construction exploits the fact, already mentioned in Section 4.3.1, that every Presburger formula is equivalent to a TR-constraint; in other words, the Presburger-definable predicates are the smallest set of predicates containing all threshold and remainder predicates, and closed under boolean operations [13]. (Recall that, by definition, threshold and remainder predicates are the predicates ${\mathbb {N}}^k \rightarrow \{0,1\}$ expressible by the threshold and remainder constraints introduced in Definitions 7 and 9, respectively.) We show that all threshold and remainder predicates can be computed by protocols in ${ WS}^3$, and that ${ WS}^3$ is closed under negation and conjunction. As a consequence, we obtain that ${ WS}^3$ is as expressive as ${ WS}$, the class of all well-specified protocols.

### Threshold protocol

We describe the protocol given in [3] to compute the threshold predicate $\sum _{i=1}^k a_i x_i < c$, first informally, and then formally. Define

$$\begin{aligned} v_\text {max}{\mathop {=}\limits ^{\text {def}}}\max (|a_1|, |a_2|, \ldots , |a_k|, |c|+1). \end{aligned}$$

States are triples of the form $(\ell , n, o)$, where $\ell$ and *o* are Booleans, and $n \in [-v_\text {max}, v_\text {max}]$. Let us first describe the intended meaning of *n*. Intuitively, if an agent is in state $(\ell , n, o)$, then *n* indicates its current wealth (which can be negative). Observe that the wealth of an agent always lies in the interval $[-v_\text {max}, v_\text {max}]$. Initially, agents in state $I(x_i)$ have wealth $a_i$. So the goal of the protocol is to decide whether the global wealth $\sum _{i=1}^k a_i x_i$ owned collectively by all agents is below the threshold *c*. Let us now consider the components $\ell$ and *o* of a state $(\ell , n, o)$. Component *o* indicates the current opinion of the agent, i.e., whether it currently believes the global wealth is below *c*. Component $\ell$ indicates whether the agent is active or passive. Initially all agents are active, and their opinion is given by their own wealth. For example, if an agent has wealth 3 and $c=2$, then its current opinion is that the global wealth is not below *c*.

Interactions only take place between an active agent and another agent, which may be active or not. The two agents update their states as follows:

- The first agent remains active, and the second becomes (or remains) passive.
- The two agents compute their joint wealth, and update their opinions according to it. For example, if the agents have wealths $-2$ and 3, and $c=2$, then after the interaction both agents believe the global wealth is below *c*.
- The two agents redistribute their joint wealth as follows. The second agent receives an amount whose absolute value is as close to 0 as possible (while respecting the constraint that individual wealths are in $[-v_\text {max}, v_\text {max}]$), and the first agent receives the rest. For example, if the wealth interval is $[-3, 3]$ and the agents have individual wealths $-1$ and 3, then after redistribution their wealths become 2 and 0; if they are $-2$ and $-2$, they become $-3$ and $-1$; and if they are 0 and 3, they become 3 and 0.

Intuitively, the protocol works because eventually one single agent remains active, and its wealth stabilizes to a value *n* satisfying the following property: if the global wealth is in the interval $[-v_\text {max}, v_\text {max}]$, then *n* is the global wealth, and if the global wealth is larger than $v_\text {max}$ (resp. smaller than $-v_\text {max}$), then $n = v_\text {max}$ (resp. $n = -v_\text {max}$). In all cases, the opinion of this agent eventually stabilizes to the correct answer to the question whether the global wealth is below *c*, and the agent eventually changes the opinion of all other agents to this value. More details can be found in [3].

Formally, define

$$\begin{aligned} f(m, n)&{\mathop {=}\limits ^{\text {def}}}\max (-v_\text {max}, \min (v_\text {max}, m + n)), \\ g(m, n)&{\mathop {=}\limits ^{\text {def}}}(m + n) - f(m, n), \\ b(m, n)&{\mathop {=}\limits ^{\text {def}}}(f(m, n) < c). \end{aligned}$$

The protocol is ${\mathcal {P}}_{\text {thr}} {\mathop {=}\limits ^{\text {def}}}(Q, T, X, I, O)$, where

$$\begin{aligned} Q&{\mathop {=}\limits ^{\text {def}}}\{0, 1\} \times [-v_\text {max}, v_\text {max}] \times \{0, 1\}, \\ X&{\mathop {=}\limits ^{\text {def}}}\{x_1, x_2, \ldots , x_k\}, \\ I(x_i)&{\mathop {=}\limits ^{\text {def}}}(1, a_i, a_i < c) \text { for every } i \in [k], \\ O(\ell , n, o)&{\mathop {=}\limits ^{\text {def}}}o \text { for every state } (\ell , n, o), \end{aligned}$$

and *T* contains

$$\begin{aligned} (1, n, o), (l, n', o')&\mapsto (1, f(n, n'), b(n, n')), (0, g(n, n'), b(n, n')) \end{aligned}$$

for every $n, n' \in [-v_\text {max}, v_\text {max}]$, $\ell , o, o' \in \{0,1\}$. Intuitively, an agent in state $(\ell , n, o)$ has value *n* (which can be positive or negative), opinion *o*, and is a leader if and only if $\ell = 1$; it is useful to think of *n* as the current wealth of the agent. The wealth of an agent lies always in the interval $[-v_\text {max}, v_\text {max}]$. Initially, agents in state $I(x_i)$ have wealth $a_i$. So, intuitively, the goal of the protocol is to decide if the total wealth $\sum _{i=1}^k a_i x_i$ own by all agents together is below a threshold *c*.

Let $\text {val}(q) {\mathop {=}\limits ^{\text {def}}}n$ for every state $q = (\ell , n, o) \in Q$, and let $\text {val}(C) {\mathop {=}\limits ^{\text {def}}}\sum _{q \in Q} C(q) \cdot \text {val}(q)$ for every configuration $C \in \text {Pop}(Q)$.

#### Proposition 11

For every $C, C' \in \text {Pop}(Q)$ and $\mathbf {x} : T \rightarrow {\mathbb {N}}$, if $(C, C', \mathbf {x})$ is a solution to the flow equations, then $\text {val}(C) = \text {val}(C')$.

#### Proof

Assume $(C, C', \mathbf {x})$ is a solution to the flow equations. For all $m, n \in [-v_\text {max}, v_\text {max}]$, we have $g(m, n) + f(m, n) = m + n$. Therefore, $\text {val}({\text {pre}(t)}) = \text {val}({\text {post}(t)})$ for every $t \in T$. This implies:

$$\begin{aligned} \text {val}(C')&= \sum _{q \in Q} \left( C(q) + \sum _{t \in T} \mathbf {x}(t) \cdot ({\text {post}(t)}(q) - {\text {pre}(t)}(q))\right) \cdot \text {val}(q) \\&= \text {val}(C) + \sum _{q \in Q} \sum _{t \in T} \mathbf {x}(t) \cdot ({\text {post}(t)}(q) - {\text {pre}(t)}(q)) \cdot \text {val}(q) \\&= \text {val}(C) + \sum _{t \in T} \mathbf {x}(t) \cdot \left[ \sum _{q \in Q} {\text {post}(t)}(q) \cdot \text {val}(q) - \sum _{q \in Q} {\text {pre}(t)}(q) \cdot \text {val}(q)\right] \\&= \text {val}(C) + \sum _{t \in T} \mathbf {x}(t) \cdot \left[ \text {val}({\text {post}(t)}) - \text {val}({\text {pre}(t)})\right] \\&= \text {val}(C). \end{aligned}$$

$$\square $$

#### Proposition 12

Let $C, C' \in \text {Pop}(Q)$ be terminal configurations that contain a leader. Both *C* and $C'$ are consensus configurations. Moreover, if $\text {val}(C) = \text {val}(C')$, then $O(C) = O(C')$.

#### Proof

We prove the first claim for *C*. The argument is identical for $C'$. Suppose that *C* is not a consensus configuration. Let $(1, m, o) \in \llbracket C\rrbracket$ be a leader of *C*. Since *C* is not a consensus configuration, there exists $(\ell , n, \lnot o) \in \llbracket C\rrbracket$. Therefore, the following transition *t* is enabled at *C*:

$$\begin{aligned} (1, m, o), (\ell , n, \lnot o) \mapsto (1, f(m, n), b(m, n)), (0, g(m, n), b(m, n)). \end{aligned}$$

Moreover, *t* is non silent which contradicts the fact that *C* is terminal. Thus, *C* is a consensus configuration.

Assume that $\text {val}(C) = \text {val}(C')$. Suppose that $O(C) \ne O(C')$ for the sake of contradiction. Without loss of generality, we may assume that $O(C) = 1$ and $O(C') = 0$. Let $p_C, p_{C'} \in Q$ be respectively leaders of *C* and $C'$. We have $\text {val}(p_C)< c < v_\text {max}$ and $\text {val}(p_{C'}) \ge c > -v_\text {max}$. We claim that

4 $$\begin{aligned} \text {val}(p_C) \ge \text {val}(C) \text { and } \text {val}(p_{C'}) \le \text {val}(C'). \end{aligned}$$

To see that the claim holds, suppose that $\text {val}(p_C) < \text {val}(C)$. There exists some $q_C \in \llbracket C\rrbracket$ such that $\text {val}(q_C) > 0$. Since $\text {val}(p_C) < v_\text {max}$, some part of the value of $q_C$ can be transferred to $p_C$, i.e. there exists a non silent transition $t \in T$ with ${\text {pre}(t)} =$, which contradicts that *C* is terminal. Thus, $\text {val}(p_C) \ge \text {val}(C)$ holds. The case $\text {val}(p_{C'}) \le \text {val}(C')$ follows by a similar argument.

Now, by (4) we have $\text {val}(C) \le \text {val}(p_C) < c$ and $\text {val}(C') \ge \text {val}(p_{C'}) \ge c$ which is a contradiction since $\text {val}(C) = \text {val}(C')$. Therefore, $O(C) = O(C')$. $\square$

#### Proposition 13

$${\mathcal {P}}_{\text {thr}}$$ satisfies StrongConsensus.

#### Proof

Suppose for the sake of contradiction that ${\mathcal {P}}_{\text {thr}}$ does not satisfy StrongConsensus. There are two cases to consider.

- There exist $C, C' \in \text {Pop}(Q)$ such that , *C* is initial, $C'$ is terminal and $C'$ is not a consensus configuration. Since *C* is initial, it contains a leader. It is readily seen that the set of leaders forms a *U*-trap for every $U \subseteq T$, which implies that $C'$ contains a leader as $(C, C', \mathbf {x})$ satisfies the *U*-trap constraints for all *U*. By Proposition 12, $C'$ is a consensus configuration, which is a contradiction.
- There exist $C_0, C, C' \in \text {Pop}(Q)$ and $\mathbf {x}, \mathbf {x}' : T \rightarrow {\mathbb {N}}$ such that , , $C_0$ is initial, *C* and $C'$ are terminal consensus configurations, and $O(C) \ne O(C')$. Note that $(C_0, C, \mathbf {x})$ and $(C_0, C', \mathbf {x}')$ both satisfy the flow equations. Therefore, by Proposition 11, $\text {val}(C) = \text {val}(C_0) = \text {val}(C')$. Again, since $C_0$ is initial, it contains a leader, which implies that both *C* and $C'$ contain a leader. Since $\text {val}(C) = \text {val}(C')$, Proposition 12 yields $O(C) = O(C')$ which is a contradiction.

$$\square $$

#### Proposition 14

$${\mathcal {P}}_{\text {thr}}$$ satisfies LayeredTermination.

#### Proof

Assume $c > 0$. The case where $c \le 0$ follows by a symmetric argument. Let $L_0 {\mathop {=}\limits ^{\text {def}}}\{(1, x, 0) : c \le x \le v_\text {max}\}$ and $N_1 {\mathop {=}\limits ^{\text {def}}}\{(0, 0, 1)\}$. We claim that the following ordered partition satisfies layered termination:

$$\begin{aligned} T_1&{\mathop {=}\limits ^{\text {def}}}\{t \in T : {\text {pre}(t)} \ne \text { for all } q \in L_0, r \in N_1\}, \\ T_2&{\mathop {=}\limits ^{\text {def}}}T \setminus T_1. \end{aligned}$$

We first show that every execution of ${\mathcal {P}}_{\text {thr}}[T_1]$ is fair. For the sake of contradiction, assume this is not the case. There exists a non silent execution $C_0 \xrightarrow {t_1} C_1 \xrightarrow {t_2} \cdots$ where $C_0, C_1, \ldots \in \text {Pop}(Q)$ and $t_1, t_2, \ldots \in T_1$. For every $i \in {\mathbb {N}}$, let

$$\begin{aligned} \text {leaders}(C_i)&{\mathop {=}\limits ^{\text {def}}}\{q \in \llbracket C_i\rrbracket : q \text { is a leader}\},\\ \text {nonleaders}(C_i)&{\mathop {=}\limits ^{\text {def}}}\{q \in \llbracket C_i\rrbracket : q \text { is not a leader}\}, \\ \text {num}\text {-}\text {leaders}(C_i)&{\mathop {=}\limits ^{\text {def}}}\sum _{q \in \text {leaders}(C_i)} C_i(q), \\ z_i&{\mathop {=}\limits ^{\text {def}}}\sum _{q \in \text {nonleaders}(C_i)} C_i(q) \cdot |\text {val}(q)|. \end{aligned}$$

Since no transition increases the number of leaders, there exists some $n_1 \in {\mathbb {N}}$ such that $\text {num}\text {-}\text {leaders}(C_i) = \text {num}\text {-}\text {leaders}(C_{i+1})$ for all $i \ge n_1$. Moreover, generalizing an observation made in [3], we have that $z_i < z_{i+1}$ implies $\text {num}\text {-}\text {leaders}(C_i) \ne \text {num}\text {-}\text {leaders}(C_{i+1})$, which entails $z_{n_1} \ge z_{n_1 + 1} \ge \ldots$. Therefore, there exists $n_2 \in {\mathbb {N}}$ such that $z_i = z_{n_2}$ for every $i \ge n_2$.

Let $L_\text {err} {\mathop {=}\limits ^{\text {def}}}\{(1, x, b) : -v_\text {max}\le x \le v_\text {max}, b \not = (x < c)\}$ be the set of leaders whose opinion is inconsistent with their value. Since no transition of ${\mathcal {P}}_{\text {thr}}$ produces states from $L_\text {err}$, transitions involving a state from $L_\text {err}$ can only be taken in finitely many steps. More formally, there exists $n_3 \in {\mathbb {N}}$ such that $\llbracket {\text {pre}(t_i)}\rrbracket \cap L_\text {err} = \emptyset$ for every $i > n_3$. Let $n {\mathop {=}\limits ^{\text {def}}}\max (n_1, n_2, n_3)$. Any non silent transition $t_i$ such that $i > n$ must be of the form:

$$\begin{aligned} (1, x, 1), (0, 0, 0)&\mapsto (1, x, 1), (0, 0, 1) \end{aligned}$$

for some $x < c$, as otherwise one of the above observations would be violated. But such transitions set the opinion of non leaders to 1, which can only occur for finitely many steps. Therefore, there exists $n' \ge n$ such that every transition enabled in $C_{n'}$ is silent. This is a contradiction.

It is readily seen that any execution of ${\mathcal {P}}_{\text {thr}}[T_2]$ is silent since each transition of $T_2$ is of the form:

$$\begin{aligned} (1, x, 0), (0, 0, 1) \mapsto (1, x, 0), (0, 0, 0) \end{aligned}$$

for some $c \le x \le v_\text {max}$. Therefore, it remains to prove that ${\mathcal {P}}_{\text {thr}}[T_2]$ is $T_1$-dead. Let $C \in \text {Pop}(Q)$ be a $T_1$-dead configuration. For the sake of contradiction, suppose there exists $w \in T_2^+$ and $C' \in \text {Pop}(Q)$ such that $C \xrightarrow {w} C'$ and $C'$ enables some non silent transition $t \in T_1$. Since *C* is $T_1$-dead, transition *t* must be of the form

$$\begin{aligned} (1, y, 1), (0, 0, 0) \mapsto (1, y, 1), (0, 0, 1) \end{aligned}$$

for some $y < c$. Moreover, (1, *y*, 1) already appeared in *C*. This means that *C* contains one leader of opinion 0, and one leader of opinion 1. Therefore, *C* is not $T_1$-dead, which is a contradiction. $\square$

### Remainder protocol

We give a protocol for the remainder predicate

$$\begin{aligned} \sum _{i=1}^k a_i x_i \equiv c \pmod {m}. \end{aligned}$$

The protocol is ${\mathcal {P}}_{\text {rmd}} = (Q, T, X, I, O)$, where

$$\begin{aligned} Q&{\mathop {=}\limits ^{\text {def}}}[0, m) \cup \{\text {true}, \text {false}\} \\ X&{\mathop {=}\limits ^{\text {def}}}\{x_1, x_2, \ldots , x_k\} \\ I(x_i)&{\mathop {=}\limits ^{\text {def}}}a_i \bmod m \text { for every } i \in [k] \\ O(q)&{\mathop {=}\limits ^{\text {def}}}{\left\{ \begin{array}{ll} 1 \text { if } q \in \{c, \text {true}\} \\ 0 \text { otherwise } \end{array}\right. } \end{aligned}$$

and where *T* contains the following transitions for all $n, n' \in [0, m)$ and $b \in \{\text {false}, \text {true}\}$:

$$\begin{aligned} (n, n')&\mapsto (n + n' \bmod m, n + n' \bmod m = c) \quad \text{ and } \\ (n, b)&\mapsto (n, n = c). \end{aligned}$$

Intuitively, the protocol works as follows. Each agent initially holds a numerical value. When two agents interact, one of them stores the sum of their values modulo *m*, and the other agent becomes passive. Eventually, one numerical value remains, and passive agents are converted to *true* or *false* depending on whether this value equals *c*.

Let $\text {val}(C) {\mathop {=}\limits ^{\text {def}}}\left( \sum _{n \in [0, m)} C(n) \cdot n \right) \bmod m$ for every $C \in \text {Pop}(Q)$.

#### Proposition 15

$${\mathcal {P}}_{\text {rmd}}$$ satisfies StrongConsensus.

#### Proof

For every $C, C' \in \text {Pop}(Q)$, we claim that:

- if $(C, C', \mathbf {x})$ is a solution to the flow equations for some $\mathbf {x} : T \rightarrow {\mathbb {N}}$, then $\text {val}(C) = \text {val}(C')$.
- if $C, C' \in \text {Pop}(Q)$ are terminal configuration that contain a numerical value, then both *C* and $C'$ are consensus configurations, and if $\text {val}(C) = \text {val}(C')$, then $O(C) = O(C')$.

The proof of these two claims follows from the definition of ${\mathcal {P}}_{\text {rmd}}$ as in the case of the threshold protocol.

Suppose for the sake of contradiction that ${\mathcal {P}}_{\text {rmd}}$ does not satisfy StrongConsensus. There are two cases to consider.

- There exist $C, C' \in \text {Pop}(Q)$ such that , *C* is initial, $C'$ is terminal and $C'$ is not a consensus configuration. Since $C_0$ is initial, it only contains numerical values. Since numerical values form a *U*-trap for every $U \subseteq T$, *C* contains a numerical value. By (b), *C* is a consensus configuration, which is a contradiction.
- There exist $C_0, C, C' \in \text {Pop}(Q)$ and $\mathbf {x}, \mathbf {x}' : T \rightarrow {\mathbb {N}}$ such that , , $C_0$ is initial, *C* and $C'$ are terminal consensus configurations, and $O(C) \ne O(C')$. Note that $(C_0, C, \mathbf {x})$ and $(C_0, C', \mathbf {x}')$ both satisfy the flow equations. Therefore, by (a), $\text {val}(C) = \text {val}(C_0) = \text {val}(C')$. Again, since $C_0$ is initial, it contains a numerical value, which implies that both *C* and $C'$ contain a numerical value. Since $\text {val}(C) = \text {val}(C')$, (b) yields $O(C) = O(C')$ which is a contradiction.

$$\square $$

#### Proposition 16

$${\mathcal {P}}_{\text {rmd}}$$ satisfies LayeredTermination.

#### Proof

We claim that the following ordered partition satisfies layered termination:

$$\begin{aligned} T_1&{\mathop {=}\limits ^{\text {def}}}\{t \in T : {\text {pre}(t)} = \text { for some } q \in [0, m), r \in ([0, m) \cup \{\text {false}\})\} \\ T_2&{\mathop {=}\limits ^{\text {def}}}\{t \in T : {\text {pre}(t)} = \text { for some } q \in [0, m)\} \end{aligned}$$

We first show that every execution of ${\mathcal {P}}_{\text {rmd}}[T_1]$ is silent. For the sake of contradiction, assume it is not the case. There exists a non silent execution $C_0 \xrightarrow {t_1} C_1 \xrightarrow {t_2} \cdots$ where $C_0, C_1, \ldots \in \text {Pop}(Q)$ and $t_1, t_2, \ldots \in T_1$. For every $i \in {\mathbb {N}}$, let $\text {numerical}(C_i) {\mathop {=}\limits ^{\text {def}}}\sum _{n \in [0, m)} C_i(n)$. It is readily seen that $\text {numerical}(C_0) \ge \text {numerical}(C_1) \ge \cdots$. Therefore, there exists $\ell \in {\mathbb {N}}$ such that $\text {numerical}(C_i) = \text {numerical}(C_{i-1})$ for every $i > \ell$. This implies that, for every $i > \ell$, if $t_i$ is non silent, then it is of the form $(n, \text {false}) \mapsto (n, \text {true})$ for some $n \in [0, m)$. But, these non silent transitions can only occur for a finite amount of steps, which is a contradiction.

It is readily seen that every execution of ${\mathcal {P}}_{\text {rmd}}[T_2]$ is silent since non silent transitions of $T_2$ are all of the form $(n, \text {true}) \mapsto (n, \text {false})$ for some $n \in [0, m)$. Therefore, it remains to prove that ${\mathcal {P}}_{\text {rmd}}[T_2]$ is $T_1$-dead. Let $C \in \text {Pop}(Q)$ be a $T_1$-dead configuration. For the sake of contradiction, suppose there exists $w \in T_2^+$ and $C' \in \text {Pop}(Q)$ such that $C \xrightarrow {w} C'$ and $C'$ enables some non silent transition $t \in T_1$. We have $C(\text {true}) > 0$ and $C(n) > 0$ for some $n \in [0, m)$ such that $O(n) = 0$. Moreover, since *C* is $T_1$-dead, $\text {numerical}(C) = 1$. Therefore *t* must be of the form $(n, \text {false}) \mapsto (n, \text {false})$. We obtain a contradiction since *t* is non silent. $\square$

### Negation and conjunction

Let ${\mathcal {P}}_1 = (Q_1, T_1, X, I_1, O_1)$ and ${\mathcal {P}}_2 = (Q_2, T_2, X, I_2, O_2)$ be ${ WS}^3$-protocols computing predicates $\varphi _1$ and $\varphi _2$ respectively. We may assume that ${\mathcal {P}}_1$ and ${\mathcal {P}}_2$ are defined over identical input alphabet *X*, for we can always extend the input domain of threshold/remainder predicates by variables with coefficients of value zero. The predicate $\lnot \varphi _i$ can be computed by replacing $O_i$ by the new output function $O_i'$ such that $O_i'(q) {\mathop {=}\limits ^{\text {def}}}\lnot O_i(q)$ for every $q \in Q_i$. To compute $\varphi _1 \wedge \varphi _2$, we build an asynchronous product where steps of ${\mathcal {P}}_1$ and ${\mathcal {P}}_2$ can be executed independently.

For every transition $t = (q, r) \mapsto (q', r')$, and every pair of states (*p*, *s*), let $t \mathbin {\otimes }(p,s)$ denote the transition lifted to (*p*, *s*):

$$\begin{aligned} ((q, p), (r, s)) \mapsto ((q', p), (r', s)) \end{aligned}$$

Similarly let $(p, s) \mathbin {\otimes }t$ denote the lifted transition

$$\begin{aligned} ((p, q), (s, r)) \mapsto ((p, q'), (s, r')). \end{aligned}$$

#### Definition 10

The *conjunction* of ${\mathcal {P}}_1$ and ${\mathcal {P}}_2$ is defined as the population protocol ${\mathcal {P}}{\mathop {=}\limits ^{\text {def}}}(Q, S, I, X, O)$ such that $Q {\mathop {=}\limits ^{\text {def}}}Q_1 \times Q_2$, $S {\mathop {=}\limits ^{\text {def}}}S_1 \cup S_2$, $I(X) {\mathop {=}\limits ^{\text {def}}}(I_1(X), I_2(X))$ and $O(p, q) {\mathop {=}\limits ^{\text {def}}}O_1(p) \wedge O_2(q)$ where

$$\begin{aligned} S_1&{\mathop {=}\limits ^{\text {def}}}\{ t \mathbin {\otimes }(q, r): t \in T_1, (q, r) \in Q_2\times Q_2\},\\ S_2&{\mathop {=}\limits ^{\text {def}}}\{(q, r) \mathbin {\otimes }t : t \in T_2, (q, r) \in Q_1 \times Q_1\}. \end{aligned}$$

In the rest of this section, we show that the conjunction of two ${ WS}^3$ protocols remains in ${ WS}^3$. While the proof is relatively simple, it first requires us to introduce technical lemmas that relate the product of two protocols with projections onto these protocols.

Let $i \in \{1, 2\}$. The *projection* of $q \in Q$ onto $Q_i$ is the state $\pi _i(q) {\mathop {=}\limits ^{\text {def}}}q_i$ where $q = (q_1, q_2)$. The *projection* of $t \in S_i$ on $T_i$ is the transition $\pi _i(t) {\mathop {=}\limits ^{\text {def}}}(\pi _i(p), \pi _i(q), \pi _i(p'), \pi _i(q'))$ where $t = (p, q, p', q')$. We lift projections to $\text {Pop}(Q)$ and $S \rightarrow {\mathbb {N}}$ as follows. For every $C \in \text {Pop}(Q)$ and $\mathbf {x} : S \rightarrow {\mathbb {N}}$, the *projections* $\pi _i(C) \in \text {Pop}(Q_i)$ and $\pi _i(\mathbf {x}) : T_i \rightarrow {\mathbb {N}}$ are respectively the configuration and mapping such that

$$\begin{aligned} \pi _i(C)(q)&{\mathop {=}\limits ^{\text {def}}}\sum _{\begin{array}{c} r \in Q \\ \pi _i(r) = q \end{array}} C(r) \text { for every } q \in Q_i\quad \text { and } \\ \pi _i(\mathbf {x})(t)&{\mathop {=}\limits ^{\text {def}}}\sum _{\begin{array}{c} s \in S_i \\ \pi _i(s) = t \end{array}} \mathbf {x}(s) \text { for every } t \in T_i. \end{aligned}$$

Let $\mathbf{I} _{{\mathcal {P}}} \in {\mathbb {N}}^{Q \times T}$ be the matrix such that $\mathbf{I} _{{\mathcal {P}}}(q, t) {\mathop {=}\limits ^{\text {def}}}{\text {post}(t)}(q) - {\text {pre}(t)}(q)$ for every $q \in Q$ and $t \in T$. It is readily seen that $(C, C', \mathbf {x})$ satisfies the flow equations if and only if $C' = C + \mathbf{I} _{{\mathcal {P}}} \cdot \mathbf {x}$. The same holds for the matrices $\mathbf{I} _{{\mathcal {P}}_1}$ and $\mathbf{I} _{{\mathcal {P}}_2}$ defined similarly for ${\mathcal {P}}_1$ and ${\mathcal {P}}_2$. The following holds:

#### Proposition 17

For every $i \in \{1, 2\}$, $C, C' \in \text {Pop}(Q)$ and $\mathbf {x} \in S \rightarrow {\mathbb {N}}$ we have:

- $$\pi _i(C + C') = \pi _i(C) + \pi _i(C')$$, and
- $$\pi _i\left( \mathbf{I} _{{\mathcal {P}}} \cdot \mathbf {x} \right) = \mathbf{I} _{{\mathcal {P}}_i} \cdot \pi _i(\mathbf {x})$$.

#### Proof

For every $q \in Q$, we have

$$\begin{aligned} \pi _i(C + C')(q)&= \sum _{\begin{array}{c} r \in Q \\ \pi _i(r) = q \end{array}} (C + C')(r)&\text {(by def. of } \pi _i{)} \\&= \sum _{\begin{array}{c} r \in Q \\ \pi _i(r) = q \end{array}} C(r) + C'(r) \\&= \sum _{\begin{array}{c} r \in Q \\ \pi _i(r) = q \end{array}} C(r) + \sum _{\begin{array}{c} r \in Q \\ \pi _i(r) = q \end{array}} C'(r) \\&= \pi _i(C) + \pi _i(C')&\text {(by def. of } \pi _i{)}. \end{aligned}$$

This shows (a). Let us now prove (b). Let $i \in \{1, 2\}$ and $q \in Q_i$. By definition of *S*, we have

5 $$\begin{aligned} \sum _{\begin{array}{c} r \in Q \\ \pi _i(r) = q \end{array}} \mathbf{I} _{{\mathcal {P}}}(r, t)&= 0&\text { for every } t \in S \setminus S_i, \end{aligned}$$

6 $$\begin{aligned} \sum _{\begin{array}{c} r \in Q \\ \pi _i(r) = q \end{array}} \mathbf{I} _{{\mathcal {P}}}(r, t)&= \mathbf{I} _{{\mathcal {P}}_i}(q, \pi _i(t))&\text { for every } t \in S_i. \end{aligned}$$

Informally, (5) states that although the effect $\mathbf{I} _{{\mathcal {P}}}(r, t)$ may be nonzero for a fixed state $r \in Q$, the *overall* effect of *t* cancels out to zero around a state of ${\mathcal {P}}_i$, since transition $t \in S_{1-i}$ leaves the states of ${\mathcal {P}}_i$ untouched. For example, consider the specific case of a transition

$$\begin{aligned} t = (q_1, q_1) \otimes \left( (q_2, q_2) \mapsto (q_2', q_2')\right) \text { from } S_2 \text { with } q_2 \ne q_2'. \end{aligned}$$

Let $r {\mathop {=}\limits ^{\text {def}}}(q_1, q_2)$ and $r' {\mathop {=}\limits ^{\text {def}}}(q_1, q_2')$. We have $\mathbf{I} _{{\mathcal {P}}}(r, t) + \mathbf{I} _{{\mathcal {P}}}(r', t) = -2 + 2 = 0$.

Similarly, (6) states that the overall effect of a transition $t \in S_i$ preserves the effect of its counterpart $\pi _i(t) \in T_i$ around a state of ${\mathcal {P}}_i$.

Therefore, by exploiting (5) and (6), we obtain:

$$\begin{aligned} \pi _i(\mathbf{I} _{{\mathcal {P}}} \cdot \mathbf {x})(q)&= \sum _{\begin{array}{c} r \in Q \\ \pi _i(r) = q \end{array}} (\mathbf{I} _{{\mathcal {P}}} \cdot \mathbf {x})(r)&\text {(by def. of } \pi _i{)} \\&= \sum _{\begin{array}{c} r \in Q \\ \pi _i(r) = q \end{array}} \sum _{s \in S} \mathbf{I} _{{\mathcal {P}}}(r, s) \cdot \mathbf {x}(s) \\&= \sum _{s \in S} \mathbf {x}(s) \cdot \sum _{\begin{array}{c} r \in Q \\ \pi _i(r) = q \end{array}} \mathbf{I} _{{\mathcal {P}}}(r, s) \\&= \sum _{s \in S_i} \mathbf {x}(s) \cdot \sum _{\begin{array}{c} r \in Q\\ \pi _i(r) = q \end{array}} \mathbf{I} _{{\mathcal {P}}}(r, s)&\text {(by}~(5){)} \\&= \sum _{s \in S_i} \mathbf {x}(s) \cdot \mathbf{I} _{{\mathcal {P}}_i}(q, \pi _i(s))&\text {(by}~(6){)} \\&= \sum _{t \in T_i} \mathbf{I} _{{\mathcal {P}}_i}(q, t) \cdot \sum _{\begin{array}{c} s \in S_i \\ \pi _i(s) = t \end{array}} \mathbf {x}(s) \\&= \sum _{t \in T_i} \mathbf{I} _{{\mathcal {P}}_i}(q, t) \cdot \pi _i(\mathbf {x})(t)&\text {(by def. of } \pi _i{)} \\&= (\mathbf{I} _{{\mathcal {P}}_i} \cdot \pi _i(\mathbf {x}))(q). \end{aligned}$$

$$\square $$

#### Proposition 18

For every $C, C' \in \text {Pop}(Q)$, $\mathbf {x} : S \rightarrow {\mathbb {N}}$ and $i \in \{1, 2\}$, if , then .

#### Proof

*Flow equations:* We have $C' = C + \mathbf{I} _{{\mathcal {P}}} \cdot \mathbf {x}_j$. Therefore, for every $i \in \{1, 2\}$,

$$\begin{aligned} \pi _i(C')&= \pi _i(C + \mathbf{I} _{{\mathcal {P}}} \cdot \mathbf {x}) \\&= \pi _i(C) + \pi _i(\mathbf{I} _{{\mathcal {P}}} \cdot \mathbf {x})&\text { (by Proposition }~17\text {(a))} \\&= \pi _i(C) + \mathbf{I} _{{\mathcal {P}}_i} \cdot \pi _i(\mathbf {x})&\text { (by Proposition }~17\text {(b))}. \end{aligned}$$

*Trap constraints:* For the sake of contradiction, suppose there exists $i \in \{1, 2\}$ such that a *U*-trap constraint is violated by $(\pi _i(C), \pi _i(C'), \pi _i(\mathbf {x}))$ for some $P \subseteq Q_i$. As both cases are symmetric, we may assume without loss of generality that $i = 1$. We have

7 $$\begin{aligned} {^\bullet P} \cap \llbracket \pi _1(\mathbf {x})\rrbracket \ne \emptyset ,\ {P^\bullet } \cap \llbracket \pi _1(\mathbf {x})\rrbracket \subseteq {^\bullet P}\ \text { and }\ C'(P) = 0 \end{aligned}$$

Let $R {\mathop {=}\limits ^{\text {def}}}P \times Q_2$. By definition of projections, we have

8 $$\begin{aligned} \pi _1(C')(P) = 0&\iff C'(R) = 0. \end{aligned}$$

where $\pi _1(C')(P)$ is the total number of agents the configuration $\pi _1(C')$ puts in *P*. We claim that

9 $$\begin{aligned} {^\bullet R} \cap \llbracket \mathbf {x}\rrbracket&\ne \emptyset , \end{aligned}$$

10 $$\begin{aligned} {R^\bullet } \cap \llbracket \mathbf {x}\rrbracket&\subseteq {^\bullet R}. \end{aligned}$$

Observe that if these claims hold then we are done. Indeed, if (10) holds, then *R* is a $\llbracket \mathbf {x}\rrbracket$-trap, and if moreover (9) holds, then, by (8), $(\pi _i(C), \pi _i(C'), \pi _i(\mathbf {x}))$ violates the $\llbracket \mathbf {x}\rrbracket$-trap constraint for *R*.

It remains to prove the claims. For (9), let $t \in {^\bullet P} \cap \llbracket \pi _1(\mathbf {x})\rrbracket$. By assumption, such a *t* must exist. Since $t \in {^\bullet P}$, we have that $t :(p, p') \mapsto (r, r')$ with $r \in P$ or $r' \in P$. Moreover, since $t \in \llbracket \pi _1(\mathbf {x})\rrbracket$, by definition of projections there must exist some $t' \in \llbracket \mathbf {x}\rrbracket$ given by

$$\begin{aligned} \left( (p, q), (p', q') \right) \mapsto \left( (r, q), (r', q') \right) \end{aligned}$$

for some $q, q' \in Q_2$. It remains to show that $t' \in {^\bullet R}$. For this observe that, since $r \in P$ or $r' \in P$, we have that $(r, q) \in R$ or $(r', q') \in R$, and thus $t' \in {^\bullet R}$. This concludes the proof of  (9).

For  (10), let $t \in {R^\bullet } \cap \llbracket \mathbf {x}\rrbracket$. There exist $p \in P$ and $q \in Q_2$ such that $(p, q) \in {^\bullet t}$. Moreover, $\mathbf {x}(t) > 0$. We must prove $t \in {^\bullet R}$. We consider two cases

- Assume $t \in S_2$. By definition of $S_2$, *t* is of the form
  $$\begin{aligned} \left( (p, q), (p', q') \right) \mapsto \left( (p, r), (p', r') \right) \end{aligned}$$
  for some $p' \in Q_1$ and $q', r, r' \in Q_2$. In particular, we have $(p, r) \in {t^\bullet }$ which implies $t \in {^\bullet (P \times Q_2)} = {^\bullet R}$.
- Assume $t \in S_1$. Let $s {\mathop {=}\limits ^{\text {def}}}\pi _1(t)$. By definition of $S_2$, *t* is of the form
  $$\begin{aligned} \left( (p, q), (p', q') \right) \mapsto \left( (r, q), (r', q') \right) \end{aligned}$$
  where ${\text {pre}(s)} =$, ${\text {post}(s)} =$ and $q' \in Q_2$. This implies that $s \in {p^\bullet } \subseteq {P^\bullet }$. Moreover, since $t \in \llbracket \mathbf {x}\rrbracket$, we have $s \in \llbracket \pi _1(\mathbf {x})\rrbracket$. Therefore, by (7), we have $s \in {^\bullet P}$. This implies that either $r \in P$ or $r' \in P$, which in turn implies that $t \in {^\bullet R}$.

*U-Siphon constraints:* Symmetric to *U*-trap constraints. $\square$

#### Proposition 19

For every $i \in \{1, 2\}$, $C \in \text {Pop}(Q)$ and $t \in T_i$, *t* is enabled in $\pi _i(C)$ if and only if there exists $s \in S_i$ such that $\pi _i(s) = t$ and *s* is enabled in *C*.

#### Proof

We only prove the claim for $i = 1$, as the case $i = 2$ is symmetric. Let $p, q \in Q_1$ be such that ${\text {pre}(t)} =$. By definition of $\pi _1$, we have

$$\begin{aligned} \pi _1(C)(p_1)&{\mathop {=}\limits ^{\text {def}}}\sum _{p_2 \in Q_2} C(p_1, p_2), \text { and} \\ \pi _1(C)(q_1)&{\mathop {=}\limits ^{\text {def}}}\sum _{q_2 \in Q_2} C(q_1, q_2). \end{aligned}$$

This implies that

11 $$\begin{aligned} \pi _1(C) \ge \iff \exists p_2, q_2 \in Q_2 \text { s.t. } C \ge . \end{aligned}$$

$\Rightarrow$) Assume *t* is enabled in $\pi _1(C)$. By (11), $C \ge$ for some $p_2, q_2 \in Q_2$. Let

$$\begin{aligned} s&{\mathop {=}\limits ^{\text {def}}}t \mathbin {\otimes }(p_2, q_2) \end{aligned}$$

We have $s \in S_1$. Moreover, *s* is enabled at *C*.

$\Leftarrow$) Assume there exists $s \in S_1$ such that $\pi _1(s) = t$ and *s* is enabled at *C*. By definition of $S_1$,

$$\begin{aligned} s&= t \mathbin {\otimes }(p_2, q_2) \end{aligned}$$

for some $p_2, q_2 \in Q_2$. Since *s* is enabled at *C*, we have $C \ge$. By (11), this implies $\pi _1(C) \ge$, which in turn implies that *t* is enabled at $\pi _1(C)$. $\square$

#### Corollary 3

For every $C \in \text {Pop}(Q)$, if *C* is terminal in ${\mathcal {P}}$, then $\pi _1(C)$ and $\pi _2(C)$ are respectively terminal in ${\mathcal {P}}_1$ and ${\mathcal {P}}_2$.

#### Proof

Let $C \in \text {Pop}(Q)$ be such that *C* is terminal in ${\mathcal {P}}$. For the sake of contradiction, suppose there exists $i \in \{1, 2\}$ such that $\pi _i(C)$ is not terminal in ${\mathcal {P}}_i$. There exists $t \in T_i$ such that *t* is non silent and enabled in $\pi _i(C)$. By Proposition 19, there exists $s \in S_i$ such that $\pi _i(s) = t$ and *s* is enabled at *C*. We have $s = t \mathbin {\otimes }q$ for some $q \in Q_2 \times Q_2$. This implies that *s* is non silent, since *t* is non silent. We conclude that *C* is non terminal which is a contradiction. $\square$

#### Lemma 5

If ${\mathcal {P}}_1$ and ${\mathcal {P}}_2$ satisfy StrongConsensus, then ${\mathcal {P}}$ satisfies StrongConsensus.

#### Proof

We prove the contrapositive: if ${\mathcal {P}}$ does not satisfy StrongConsensus, then at least one of ${\mathcal {P}}_1$ and ${\mathcal {P}}_2$ does not satisfy StrongConsensus. Assume ${\mathcal {P}}$ does not satisfy StrongConsensus. There are two cases to consider.

- There exist $C, C' \in \text {Pop}(Q)$ such that , *C* is initial, $C'$ is a terminal non consensus configuration. Since $C'$ is a non consensus configuration, there exist $(p, q), (p', q') \in \llbracket C'\rrbracket$ such that $O_1(p) \wedge O_1(q) = O(p, q) \not = O(p', q') = O_2(p') \wedge O_2(q')$. Without loss of generality, we can assume that $O_1(p) \not = O_1(p')$. By Corollary 3, $\pi _1(C')$ is terminal in ${\mathcal {P}}_1$. Moreover, since $p, p' \in \pi _1(C')$, $\pi _1(C')$ is a non consensus configuration. Therefore, $\pi _1(C')$ is a terminal non consensus configuration of ${\mathcal {P}}_1$. Moreover, by Proposition 18 which implies that ${\mathcal {P}}_1$ does not satisfy StrongConsensus.
- There exist $C_0, C, C' \in \text {Pop}(Q)$ and $\mathbf {x}, \mathbf {x}' : T \rightarrow {\mathbb {N}}$ such that , , $C_0$ is initial, *C* and $C'$ are terminal consensus configurations, and $O(C) \ne O(C')$. Since *C* and $C'$ have different opinions, there exist $(p, q) \in \llbracket C\rrbracket$ and $(p', q') \in \llbracket C'\rrbracket$ such that $O(p, q) \not = O(p', q')$. Without loss of generality, we can assume that $O_1(p) \not = O_1(p')$. By Corollary (3), $\pi _1(C)$ and $\pi _1(C')$ are terminal in ${\mathcal {P}}_1$. Moreover, since $p \in \pi _1(C)$ and $p' \in \pi _1(C')$, $\pi _1(C)$ and $\pi _1(C')$ are terminal configuration with different consensus. Moreover, by Proposition 18,  and  which implies that ${\mathcal {P}}_1$ does not satisfy StrongConsensus.

$$\square $$

#### Proposition 20

If ${\mathcal {P}}_1$ and ${\mathcal {P}}_2$ satisfy LayeredTermination, then ${\mathcal {P}}$ satisfies LayeredTermination.

#### Proof

Let $X_1, X_2, \ldots , X_m$ and $Y_1, Y_2, \ldots , Y_n$ be ordered partitions respectively for LayeredTermination in ${\mathcal {P}}_1$ and ${\mathcal {P}}_2$. We may assume without loss of generality that $m \ge n$. For every $n < i \le m$, we define $Y_i {\mathop {=}\limits ^{\text {def}}}\emptyset$.

For every $i \in [m]$, we let

$$\begin{aligned} Z_i\ {\mathop {=}\limits ^{\text {def}}}&\{t \mathbin {\otimes }r : t \in X_i, r \in Q_2 \times Q_2\} \cup {} \\&\{r \mathbin {\otimes }t : t \in Y_i, r \in Q_1 \times Q_1\}. \end{aligned}$$

We claim that $Z_1, Z_2, \ldots , Z_m$ is an ordered partition for LayeredTermination in ${\mathcal {P}}$. Let $i \in [m]$. Let us show that every execution of ${\mathcal {P}}[Z_i]$ is silent. Suppose for the sake of contradiction that there exist $C_0, C_1, \ldots \in \text {Pop}(Q)$ and $t_0, t_1, \ldots \in Z_i$ such that $C_0 \xrightarrow {t_0} C_1 \xrightarrow {t_1} \cdots$ is non silent. There exists $j \in \{1, 2\}$ such that infinitely many non silent transitions $t_i$ belong to $S_j$. Let $i_0< i_1 < \cdots$ be all indices such that $t_{i_k} \in S_j$. We have

$$\begin{aligned} \pi _j(C_{i_0}) \xrightarrow {\pi _j(t_{i_0})} \pi _j(C_{i_1}) \xrightarrow {\pi _j(t_{i_1})} \cdots \end{aligned}$$

which is an infinite non silent execution of ${\mathcal {P}}_1[X_i]$ or ${\mathcal {P}}_2[Y_i]$ depending on *j*. This is a contradiction.

Let $W {\mathop {=}\limits ^{\text {def}}}(Z_1 \cup \cdots \cup Z_{i-1})$. Let us now prove that ${\mathcal {P}}[Z_i]$ is *W*-dead. For the sake of contradiction, assume it is not. There exist $C, C' \in \text {Pop}(Q)$, $w \in Z_i^+$ and $t \in W$ such that *C* is *W*-dead, $C \xrightarrow {w} C'$ and *t* is enabled at $C'$. We have $t \in S_j$ for some $j \in \{1, 2\}$. We may assume without loss of generality that $j = 1$. Since *C* is *W*-dead, $\pi _j(C)$ is $(X_1 \cup \cdots \cup X_{i-1})$-dead. But then, $\pi _1(C) \xrightarrow {*} \pi _1(C')$ and $t \in X_1 \cup \cdots \cup X_{i-1}$ is enabled at $C'$ which is a contradiction. $\square$

#### Corollary 4

If ${\mathcal {P}}_1$ and ${\mathcal {P}}_2$ belong to ${ WS}^3$, then ${\mathcal {P}}$ belongs to ${ WS}^3$ and is correct.

#### Proof

By Lemma 5 and Proposition 20, ${\mathcal {P}}$ belongs to ${ WS}^3$. Let $w \in \text {Pop}(X)$, $C {\mathop {=}\limits ^{\text {def}}}I(w)$, $C_1 {\mathop {=}\limits ^{\text {def}}}I_1(w)$ and $C_2 {\mathop {=}\limits ^{\text {def}}}I_2(w)$. Note that all three protocols are well-specified since they belong to ${ WS}^3$. Therefore, there exist terminal consensus configurations $C' \in \text {Pop}(Q)$, $C_1' \in \text {Pop}(Q_1)$ and $C_2' \in \text {Pop}(Q_2)$ such that $C \xrightarrow {*} C'$, $C_1 \xrightarrow {*} C_1'$ and $C_2 \xrightarrow {*} C_2'$.

We must prove that $O(C') = O_1(C_1') \wedge O_2(C_2')$. Let $j \in \{1, 2\}$. Since $C \xrightarrow {*} C'$, we have . By Proposition 18, . By definition of *I*, we have $C_j = \pi _j(C)$. Therefore, . Moreover, by Corollary 3, $\pi _j(C')$ is terminal in ${\mathcal {P}}_j$. Since ${\mathcal {P}}_j$ belongs to ${ WS}^3$, $\pi _j(C')$ is a consensus configuration such that $O_j(\pi _j(C')) = O_j(C_j')$. Altogether, we obtain

$$\begin{aligned} O(C')&= O_1(\pi _1(C')) \wedge O_2(\pi _2(C'))&\text {(by def. of } O) \\&= O_1(C_1') \wedge O_2(C_2').&\end{aligned}$$

$$\square $$

## Experimental results

We have developed a tool called Peregrine^5^ that can check whether a given protocol belongs to ${ WS}^3$ and, if so, whether it correctly computes a given predicate specified as a TR-constraint. Peregrine is implemented on top of the SMT solver Z3 [28].

Peregrine reads in a population protocol ${\mathcal {P}}= (Q, T, X, I, O)$ and constructs two sets of constraints. The first set is satisfiable if and only if LayeredTermination holds, and the second is unsatisfiable if and only if Strong-$\varphi$-Consensus holds.

For LayeredTermination, our tool Peregrine iteratively constructs constraints checking the existence of an ordered partition of size $1,$ $2,$ $\ldots ,|T|$ and decides if they are satisfiable. To check that the execution of a layer is silent, the constraints mentioned in the proof of Proposition 4 are transformed using Farkas’ lemma (see e.g. [32]) into a version that is satisfiable if and only if all the executions of the layer are silent. Also, the constraints for condition (b) of Definition 2 are added. A detailed description is given in Section 6.1.

For StrongConsensus, Peregrine initially constructs the constraints for the flow equation for three configurations $C_0, C_1, C_2$ and vectors $\mathbf {x}_1$ and $\mathbf {x}_2$, with additional constraints to guarantee that $C_0$ is initial and $C_1$ and $C_2$ are terminal.

For Strong-$\varphi$-Consensus, Peregrine constructs the constraints for the flow equation for two configurations $C_0, C_1$ and a vector $\mathbf {x}$, with additional constraints to guarantee that $C_0$ is the initial configuration for some input *X*, $C_1$ is terminal, and $\varphi (X) \ne O(C_1)$. If the constraints are unsatisfiable, then the protocol satisfies Strong-$\varphi$-Consensus. Otherwise, Peregrine searches for a *U*-trap or *U*-siphon to show that  does not hold. If, say, a *U*-siphon ${{ S}}$ is found, then Peregrine adds the constraint $C_0({{ S}}) > 0$ for all sequences requiring an agent in *S* to the set of initial constraints. This process is repeated until either the constraints are unsatisfiable and Strong-$\varphi$-Consensus is shown, or all possible *U*-traps and *U*-siphons are added, in which case Strong-$\varphi$-Consensus does not hold. We use this refinement-based approach instead of the coNP approach described in Proposition 7, as that could require a quadratic number of variables and constraints, and we generally expect to need a small number of refinement steps. The constraints for StrongConsensus are similar. A detailed description of the constraints for Strong-$\varphi$-Consensus is given in Sect. 6.2.

We evaluated Peregrine on a set of benchmarks described below. The verification times are presented in Table 1. The benchmarks are:

- The *threshold* protocols of [3] described in Section 5.
  This is an infinite family of protocols for the predicates $\sum _{i=1}^k a_ i x_i \le c$, where $a_1, \ldots , a_k, c \in \mathbb {Z}$. The states and transitions of the protocol for a given predicate depend only on $v_\text {max}{\mathop {=}\limits ^{\text {def}}}\max (|a_1|, |a_2|, \ldots , |a_k|, |c|+1)$, i.e., protocols for predicates with the same value of $v_\text {max}$ differ only on their input functions. Table 1 reports on the verification time for the predicates $\sum _{i=-v_\text {max}}^{v_\text {max}} i \cdot x_i \le 1$. We fix $c=1$ because the execution time is almost independent of *c*. The choice of the $a_i$ is the one with the longest verification time.
- The *remainder* protocols of [3] described in Section 5.
  Again, this is an infinite family of protocols for the predicates $\sum _{i=1}^k a_i x_i \equiv c \pmod {m}$. The states and transitions depend only on *m*, i.e., protocols with the same value of *m* differ only on the input function. For the same reason as above, Table 1 only reports on the verification time for the predicates $\sum _{i=1}^m i \cdot x_i \equiv 1 \pmod {m}$.
- The *average-and-conquer* majority protocols of [1].
  This is yet another infinite family of protocols, but in this case they all compute the majority predicate $x \ge y$, and differ only in their efficiency. The states and transitions depend on two parameters *m* and *d*. Since for $d > 1$ the protocols do not satisfy LayeredTermination , Peregrine cannot verify them, and so Table 1 only reports on the case $d=1$.
- Four variants of the *flock of birds*^6^ protocols, taken from [5, 8, 10, 12].
  These are four infinite families of protocols, all of them computing the predicates $x \le c$. In the first three families the protocol for $x \le c$ has *c* or $c+1$ states, whereas in the family from [8] it has ${{{\mathcal {O}}}}(\log c)$ states.
- The *broadcast* protocol of [12], the *majority* protocol of [4], and the *fast majority* protocol of [9].

**Table 1:** Results of the experimental evaluation where |*Q*| denotes the number of states, |*T*| denotes the number of non silent transitions, and the time to prove membership for ${ WS}^3$ and correctness is given in seconds. time denotes reaching the time limit of one hour

Threshold [3]				Remainder [3]				Average-and-Conquer [1]			
$v_\text {max}$	|*Q*|	|*T*|	Time	*m*	|*Q*|	|*T*|	Time	*m*	|*Q*|	|*T*|	Time
2	20	146	2.8	20	22	230	6.3	15	18	122	1.7
4	36	478	29.4	25	27	350	13.2	25	28	327	10.0
6	52	1002	190.2	30	32	495	27.7	35	38	632	45.6
8	68	1718	831.0	35	37	665	59.5	45	48	1037	192.2
9	76	2148	1945.1	40	42	860	120.3	55	58	1542	579.2
10	84	2626	2922.0	45	47	1080	308.6	65	68	2147	1615.1
11	92	3152	time	50	52	1325	time	75	78	2852	time

Flock of birds [5]				Flock of birds [10]				Flock of birds [12]			
*c*	|*Q*|	|*T*|	Time	*c*	|*Q*|	|*T*|	Time	*c*	|*Q*|	|*T*|	Time
50	50	147	21.0	20	21	210	2.9	100	101	199	63.9
100	100	297	81.9	30	31	465	10.2	200	201	399	256.2
200	200	597	383.1	40	41	820	39.4	300	301	599	678.3
300	300	897	1392.3	50	51	1275	136.8	400	401	799	1432.5
400	400	1197	2688.0	60	61	1830	435.2	500	501	999	2145.9
450	450	1347	3215.3	65	66	2145	670.6	550	551	1099	2774.8
500	500	1497	time	70	71	2485	time	600	601	1199	time

Flock of birds [8]				Non-parameterized protocols							
*c*	|*Q*|	|*T*|	Time	Protocol	|*Q*|	|*T*|	Time				
$10^{10}$	45	274	11.3	Broadcast [12]	2	1	0.2				
$10^{15}$	70	625	36.9	Majority [4]	4	4	0.2				
$10^{20}$	93	1036	104.2	Fast Majority [9]	6	10	0.3				
$10^{25}$	103	885	102.9								
$10^{30}$	137	2198	1071.5								
$10^{35}$	157	2821	2763.7								
$10^{40}$	186	4169	time								

All experiments were performed on the same machine equipped with an Intel Core i7-4810MQ CPU and 16 GB of RAM. The time limit was set to 1 hour. The results are shown in Table 1. In all cases where we terminated within the time limit, we were able to show membership for ${ WS}^3$ and correctness. Generally, showing Strong-$\varphi$-Consensus took much less time than showing LayeredTermination, except for the flock of birds protocols, where we needed linearly many *U*-traps. In comparison to only showing StrongConsensus, additionally showing Strong-$\varphi$-Consensus by additional constraints is usually faster, except for the remainder protocol.

In the forthcoming Sects. 6.1 and 6.2 , we describe in detail the constraints tested with the SMT solver in our implementation.

### Constraints for LayeredTermination

Recall that a population protocol ${\mathcal {P}}= (Q, T, X, I, O)$ satisfies LayeredTermination if there is an ordered partition $(T_1, T_2, \ldots , T_n)$ of *T* such that for every $i \in [n]$:

- every (fair or unfair) execution of ${\mathcal {P}}[T_i]$ is silent; and
- every (fair or unfair) execution of ${\mathcal {P}}[T_i]$ starting at a terminal configuration of
  ${\mathcal {P}}[T_1 \cup \cdots \cup T_{i-1}]$ contains only terminal configurations of ${\mathcal {P}}[T_1 \cup \cdots \cup T_{i-1}]$.

Given $1 \le n \le |T|$, we first derive a constraint whose solutions are the partitions $(T_1, T_2, \ldots , T_n)$ of *T* that satisfy (b) for every $i \in [n]$. Let $\textit{NS}{}$ be the set of non-silent transitions of ${\mathcal {P}}$, $U_0 = \emptyset$ and $U_i = T_1 \cup \cdots \cup T_{i-1}$ for every $i \in [n]$. Further, for every pair of transitions $s, u \in T$ let *V*(*s*, *u*) be the set of non-silent transitions $u' \in T$ such that ${\text {pre}(u')} \le {\text {pre}(s)} + ({\text {pre}(u)} \mathbin {\ominus }{\text {post}(s)})\}$. Observe that all the sets *V*(*s*, *u*) can be precomputed. Proposition 5 shows that (b) holds for a given $i \in [n]$ iff:

12 $$\begin{aligned} \text {For every } s \in T_i \text { and non-silent } u \in U_{i-1}, \text { there exists } u' \in V(s, u) \cap U_{i-1}. \end{aligned}$$

For every transition *t* let $\mathbf {b}(t)$ be an integer variable with range $\{1,2, \ldots , n\}$ and the intended meaning that $\mathbf {b}(t)=i$ iff $t \in T_i$. In other words, the valuations of the array $\mathbf {b}$ are in bijection with the partitions $(T_1, T_2, \ldots , T_n)$ of *T* (we allow some of the $T_i$ to be empty). We claim that the assignments satisfying the following constraint correspond to the partitions that satisfy condition (b) for every $i \in [n]$:

13 $$\begin{aligned} \bigwedge _{t \in T}&1 \le \mathbf {b}(t) \le n \quad \wedge \quad \bigwedge _{\begin{array}{c} s \in T \\ u \in \textit{NS}{} \end{array}} \left( \mathbf {b}(s)> \mathbf {b}(u) \rightarrow \bigvee _{u' \in V(s,u)} \mathbf {b}(s) > \mathbf {b}(u')\right) . \end{aligned}$$

Indeed, the first conjunct states that every transition is assigned to a set, and the second that (12) holds for every $i \in [n]$.

Let us now derive a constraint whose solutions are the partitions $(T_1, T_2, \ldots , T_n)$ of *T* that satisfy condition (a) for every $i \in [n]$. Fix $i \in [n]$ and let $\textit{NS}{}_i = \textit{NS}{} \cap T_i$ be the set of non-silent transitions of $T_i$. Proposition 4 shows that (a) holds for a given $i \in [n]$ iff:

14 $$\begin{aligned} \begin{aligned}&\text {There is no } \mathbf {x} :\textit{NS}{}_i \rightarrow \mathbb {Q}_{\ge 0}\text { s.t.:}\\&\quad \sum _{t \in \textit{NS}{}_i} \mathbf {x}(t) > 0 \text { and for all } q \in Q: \sum _{t \in \textit{NS}{}_i} \mathbf {x}(t) \cdot ({\text {post}(t)}(q) - {\text {pre}(t)}(q)) \ge 0. \end{aligned} \end{aligned}$$

Applying Farkas’ lemma to (14), we obtain that (a) holds for a given $i \in [n]$ iff:

15 $$\begin{aligned} \text {There is } \mathbf {y} :Q \rightarrow \mathbb {Q}_{\ge 0} \text { s.t for all } t \in \textit{NS}{}_i: \sum _{q \in Q} \mathbf {y}(q) \cdot ({\text {post}(t)}(q) - {\text {pre}(t)}(q)) < 0. \end{aligned}$$

It follows that the assignments to $\mathbf {b}$ for which the following constraint has a solution for $\mathbf {b}, \mathbf {y}_1, \ldots , \mathbf {y}_n$ correspond to the partitions that satisfy (a) for every $i \in [n]$:

16 $$\begin{aligned} \bigwedge _{\begin{array}{c} i \in [1,n] \\ t \in \textit{NS}{} \end{array}}&\left( (\mathbf {b}(t) = i) \rightarrow \left[ \bigwedge _{q \in Q} \mathbf {y}_i(q) \ge 0 \; \wedge \; \sum _{q \in Q} \mathbf {y}_i(q) \cdot ({\text {post}(t)}(q) - {\text {pre}(t)}(q)) < 0\right] \right) \end{aligned}$$

This constraint has an intuitive explanation. Let $\mathbf {b}, \mathbf {y}_1, \ldots , \mathbf {y}_n$ be a solution. Each layer $i \in [1,n]$ is given by $T_i {\mathop {=}\limits ^{\text {def}}}\{ t \in T \mid \mathbf {b}(t) = i \}$. Further, each vector $\mathbf {y}_i$ assigns a value $\mathbf {y}_i(C) {\mathop {=}\limits ^{\text {def}}}\sum _{q \in Q} \mathbf {y}_i(q) \cdot C(q)$ to each configuration *C*. For any configuration *C*, we have $\mathbf {y}_i(C) \ge 0$, and for every step $C \xrightarrow {t} C'$ where $t \in T_i$ we have $\mathbf {y}_i(C) = \mathbf {y}_i(C')$ if *t* is silent, and $\mathbf {y}_i(C) > \mathbf {y}_i(C')$ if *t* is non-silent. So the value never decreases when transitions of $T_i$ are executed, and strictly decreases when non-silent transitions occur. So $\mathbf {y}_i$ proves that every execution of ${\mathcal {P}}[T_i]$ is silent, because it can only contain finitely many occurrences of non-silent transitions.

### Constraints for Strong-$\varphi$-Consensus

As explained in the previous section, for Strong-$\varphi$-Consensus Peregrine constructs the constraints for the flow equation for two configurations $C_0, C_1$ and a vector $\mathbf {x}$, with additional constraints to guarantee that $C_0$ is the initial configuration for some input *X*, $C_1$ is terminal, and $\varphi (X) \ne O(C_1)$.

For every state $q \in Q$ let $\mathbf {c}(q)$ be a variable over ${\mathbb {N}}$. Observe that the assignments to the array $\mathbf {c}$ are in bijection with the set of configurations. The constraints for initial and terminal configurations are

$$\begin{aligned} \text {Initial}(\mathbf {c})&{\mathop {=}\limits ^{\text {def}}}\sum _{q \in I(X)} \mathbf {c}(q) \ge 2 \; \wedge \; \sum _{q \in Q \setminus I(X)} \mathbf {c}(q) = 0 \\ \text {Terminal}(\mathbf {c})&{\mathop {=}\limits ^{\text {def}}}\bigwedge _{t \in U} \bigvee _{q \in {^\bullet t}} \mathbf {c}(q) < {\text {pre}(t)}(q) \end{aligned}$$

Letting $Q_b$ be the set of states *q* with $O(q)=b$, the constraint for $\varphi (X) \ne O(C_1)$ is:

$$\begin{aligned} \text {IncorrectConsensus}_\varphi (\mathbf {c}, \mathbf {c'}) {\mathop {=}\limits ^{\text {def}}}\bigg ( \varphi ( \mathbf {c} \circ I) \wedge \sum _{q \in Q_0} \mathbf {c}(q)> 0 \bigg ) \vee \bigg ( \lnot \varphi ( \mathbf {c} \circ I) \wedge \sum _{q \in Q_1} \mathbf {c}(q) > 0 \bigg ) \end{aligned}$$

Finally, we introduce constraints related to the definition of potential reachability. For each transition $t \in T$, let $\mathbf {x}(t)$ be a variable over ${\mathbb {N}}$. We introduce a constraint expressing that the vectors $\mathbf {c}, \mathbf {c'}, \mathbf {x}$ are a solution of the flow equation:

$$\begin{aligned} \text {FlowEquation}(\mathbf {c}, \mathbf {c'}, \mathbf {x})&{\mathop {=}\limits ^{\text {def}}}\bigwedge _{q \in Q} \mathbf {c'}(q) = \mathbf {c}(q) + \sum _{t \in T} \mathbf {x}(t) \cdot ({\text {post}(t)}(q) - {\text {pre}(t)}(q)) \end{aligned}$$

and for each set $R \subseteq Q$ of states, we introduce constraints expressing conditions (b) and (c) of potential reachability (Definition 4):

$$\begin{aligned} \text {UTrap}_R(\mathbf {c}, \mathbf {c'}, \mathbf {x})&{\mathop {=}\limits ^{\text {def}}}\left( \sum _{t \in {^\bullet R}} \mathbf {x}(t)> 0 \wedge \sum _{t \in {R^\bullet } \setminus {^\bullet R}} \mathbf {x}(t) = 0 \right) \rightarrow \sum _{q \in R} \mathbf {c'}(q)> 0 \\ \text {USiphon}_S(\mathbf {c}, \mathbf {c'}, \mathbf {x})&{\mathop {=}\limits ^{\text {def}}}\left( \sum _{t \in {S^\bullet }} \mathbf {x}(t)> 0 \wedge \sum _{t \in {^\bullet S} \setminus {S^\bullet }} \mathbf {x}(t) = 0 \right) \rightarrow \sum _{q \in S} \mathbf {c}(q) > 0 \end{aligned}$$

The constraints for Strong-$\varphi$-Consensus use the variables $\mathbf {c}, \mathbf {c'} :Q \rightarrow {\mathbb {N}}$ and $\mathbf {x} :T \rightarrow {\mathbb {N}}$. For given sets ${\mathcal {R}}$ of *U*-traps and ${\mathcal {S}}$ of *U*-siphons (initially empty, and increased gradually throughout the refinement procedure described in the previous section, the constraints are:

$$\begin{aligned}&\text {FlowEquation}(\mathbf {c}, \mathbf {c'}, \mathbf {x}) \; \wedge \; \text {Initial}(\mathbf {c}) \; \wedge \; \text {Terminal}(\mathbf {c'}) \; \wedge \; \text {IncorrectConsensus}_\varphi (\mathbf {c}, \mathbf {c'}) \\&\;\wedge \; \bigwedge _{R \in {\mathcal {R}}} \text {UTrap}_R(\mathbf {c}, \mathbf {c'}, \mathbf {x}) \;\wedge \; \bigwedge _{S \in {\mathcal {S}}} \text {USiphon}_S(\mathbf {c}, \mathbf {c'}, \mathbf {x}) \end{aligned}$$

If these constraints are unsatisfiable, then Strong-$\varphi$-Consensus holds. Otherwise, we compute a solution $\texttt {c}, \texttt {c}', \texttt {x}$. We try to find an additional *U*-trap or *U*-siphon to add to ${\mathcal {R}}$ or ${\mathcal {S}}$ showing that  does not hold. The following constraints are used to find the new *U*-trap or *U*-siphon. They use the variables $\mathbf {r}: Q \rightarrow \{0,1\}$, encoding the *U*-trap or *U*-siphon $\llbracket \mathbf {r}\rrbracket$.

$$\begin{aligned} \text {FindUTrap}(\mathbf {r})&{\mathop {=}\limits ^{\text {def}}}\sum _{t \in \llbracket \texttt {x}\rrbracket } \sum _{q \in {t^\bullet }} \mathbf {r}(q)> 0 \wedge \sum _{q \in \llbracket \texttt {c'}\rrbracket } \mathbf {r}(q) = 0 \\&\wedge \bigwedge _{t \in \llbracket \texttt {x}\rrbracket } \left( \sum _{q \in {^\bullet t}} \mathbf {r}(q)> 0 \rightarrow \sum _{q \in {t^\bullet }} \mathbf {r}(q)> 0 \right) \\ \text {FindUSiphon}(\mathbf {r})&{\mathop {=}\limits ^{\text {def}}}\sum _{t \in \llbracket \texttt {x}\rrbracket } \sum _{q \in {^\bullet t}} \mathbf {r}(q)> 0 \wedge \sum _{q \in \llbracket \texttt {c}\rrbracket } \mathbf {r}(q) = 0 \\&\wedge \bigwedge _{t \in \llbracket \texttt {x}\rrbracket } \left( \sum _{q \in {t^\bullet }} \mathbf {r}(q)> 0 \rightarrow \sum _{q \in {^\bullet t}} \mathbf {r}(q) > 0 \right) \end{aligned}$$

## Conclusion and further work

We have presented ${ WS}^3$, the first class of well-specified population protocols with a membership and correctness problem of reasonable complexity (i.e. in DP) and with the full expressiveness of well-specified protocols. Previous work had shown that the membership problem for the general class of well-specified protocols is decidable, but has non-elementary complexity.

We have shown that ${ WS}^3$ is a natural class that contains many standard protocols from the literature, like flock-of-birds, majority, threshold and remainder protocols. We implemented the membership and correctness procedure for ${ WS}^3$ on top of the SMT solver Z3, yielding the first software able to automatically prove correctness of population protocols for *all* (of the infinitely many) inputs. Previous work could only prove partial correctness of protocols with at most 9 states and 28 transitions, by trying exhaustively a *finite* number of inputs [10, 12, 30, 33]. Our algorithm deals with all inputs and can handle larger protocols with up to 70 states and over 2500 transitions.

Future work will concentrate on three problems: improving the performance of our tool; extending our approach to non silent protocols; and the diagnosis problem: when a protocol does not belong to ${ WS}^3$, delivering an explanation, e.g. a non-terminating fair execution. We think that our constraint-based approach provides an excellent basis for attacking these questions.

## Figures and Tables

**Fig. 1:**
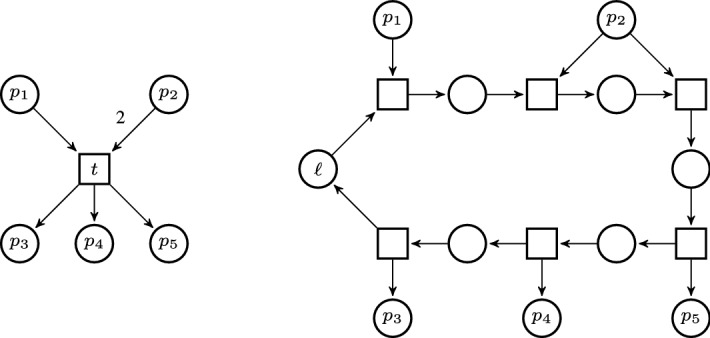
A Petri net transition (left) and its associated widget (right). Circles, squares and arcs depict respectively places, transitions and the flow function

## Appendix

### Missing Proof of Proposition 1

For the proof of Proposition 1 we need to introduce Petri nets. Intuitively, Petri nets are similar to population protocols, but their transitions can also create and destroy agents.

A *Petri net* $N = (P, T, F)$ consists of a finite set *P* of *places*, a finite set *T* of *transitions*, and a *flow function* $F :(P \times T) \cup (T\times P) \rightarrow {\mathbb {N}}$. Given a transition $t \in T$, the multiset ${\text {pre}(t)}$ of *input places* of *t* is defined by ${\text {pre}(t)}(p) = F(p,t)$, and the multiset ${\text {post}(t)}$ of *output places* by ${\text {post}(t)}(p) = F(t,p)$. A *marking* *M* of a net *N* is a multiset of places. Given a place *p*, we say that *M* puts *M*(*p*) *tokens* in *p*. A transition $t\in T$ is *enabled at* a marking *M* if ${\text {pre}(t)} \le M$. A transition *t* enabled at *M* can *fire*, yielding the marking $M' = M \mathbin {\ominus }{\text {pre}(t)} \mathbin {+}{\text {post}(t)}$. We write this fact as $M\xrightarrow {t}M'$. We extend enabledness and firing to sequences of transitions as follows. Let $\sigma =t_1\ldots t_k$ be a finite sequence of transitions $t_j\in T$. We write $M\xrightarrow {\sigma }M'$ and call it a *firing sequence* if there exists a sequence $M_0,\ldots ,M_k$ of markings such that $M=M_0\xrightarrow {t_1}M_1\cdots \xrightarrow {t_k}M_k=M'$. In that case, we say that $M'$ is *reachable from* *M* and denote by ${ Reach}(N,M)$ the set of markings reachable from $M$.

**Proposition 1** The reachability problem for Petri nets is reducible in polynomial time to the membership problem for ${ WS}^2$. In particular, membership for ${ WS}^2$ has non-elementary complexity.

#### Proof

The proof is very similar to the one of [21, Theorem 10]. However, since the proof requires small modifications at different places, we give it in full for completeness. The proof constructs a sequence of reductions from the Petri net reachability problem. Each step in the sequence transforms a problem on Petri nets into an equivalent problem closer to the model of population protocols. The first step uses a well-known result of Hack [25]. The reachability problem for Petri nets can be reduced in polynomial time to the single-place-zero-reachability problem: Given a Petri net $N_0$, a marking $M_0$, and a place ${\hat{p}}$: decide whether some marking $M \in { Reach}(N_0, M_0)$ satisfies $M({\hat{p}})=0$. We introduce a normal form for Petri nets. A Petri net $N = (P, T, F)$ is said to be in *normal form* if $F(x, y) \in \{0, 1\}$ for every $x, y \in (P \times T) \cup (T \times P)$, and every transition *t* satisfies $1 \le |{\text {pre}(t)}| \le 2$ and $1 \le |{\text {post}(t)}| \le 2$. For every Petri net $N = (P, T, F)$ and markings $M_1, M_2$, one can construct a normal form Petri net $N'=(P',T',F')$ with $P \subseteq P'$ such that $M_2$ is reachable from $M_1$ in *N* if and only if $M_2'$ is reachable from $M_1'$ in $N'$, and $M_i' = M_i + \varvec{\ell }$, where $\ell$ is a special *lock place*.

Intuitively, each transition *t* of *N* with more than two input and/or output places is simulated in $N'$ by a *widget*. The widget starts and finishes its execution by acquiring the lock and releasing it, respectively. This guarantees no two widgets are executing concurrently. When simulating transition *t*, its widget first consumes, one by one, the tokens consumed by *t* (as given by ${\text {pre}(t)}$), and then produces, one by one, the tokens produced by *t* (as given by ${\text {post}(t)}$). Figure 1 shows a transition and its widget. Observe that all transitions of the widget are in normal form.

Let $N_1$ be the result of normalizing $N_0$. Let $P_{aux}$ be the set of places of $N_1$ that are not places of $N_0$, and are different from the lock place $\ell$. The single-place-zero-reachability problem reduces to

1. Does some marking $M \in { Reach}(N_1, M_0 + \mathbf {\ell })$ satisfy $M({\hat{p}}) = 0$ and $M(P_{aux}) = 0$?
  (Observe that $M(P_{aux}) = 0$ guarantees that no widget is in the middle of its execution.)

Now we add to $N_1$ a new place $p_0$ and a new widget simulating a transition $t_0$ satisfying ${\text {pre}(t_0)} = \mathbf {p_0}$ and ${\text {post}(t_0)} = M_0 + \mathbf {\ell }$. Let the resulting net be $N_2$. Then (P1) reduces to:

(P2) Does some marking $M \in { Reach}(N_2, \mathbf {p_0})$ satisfy $M({\hat{p}}) = M(p_0) = M(P_{aux}) = 0$?

For our next step, we “reverse” $N_2$: define $N_3$ as the result of reversing all arcs of ${N}_2$, i.e., $P_3 = P_2$, $T_3 = T_2$ but $F_3(x,y) = F_2(y,x)$ for every two nodes *x*, *y*. Clearly, $N_3$ is in normal form when $N_2$ is. The problem (P2) reduces to:

(P3) Is $\mathbf {p_0} \in { Reach}(N_3, M)$ for some marking *M* of $N_3$ satisfying $M({\hat{p}}) = M(p_0) = M(P_{aux}) = 0$?

In the last step we reduce (P3) to the membership problem for ${ WS}^2$.

Let $N_3 = (P_3, T_3, F_3)$. We construct a population protocol ${\mathcal {P}}= (Q, T, X, I, O)$, defined as follows:

- $Q = P_3 \cup \{{ Fresh}, { Used}, { Collect}\}$. That is, ${\mathcal {P}}$ contains a state for each place of $N_3$, plus three auxiliary places.
- $X = Q \setminus (\{{\hat{p}}, p_0\} \cup P_{aux})$, and *I* is the identity mapping.
- $O(p_0) = 1$ and $O(q) = 0$ for every $q \ne p_0$.
- $$T = T_3' \cup T_P \cup T_s$$.

These sets are formally described below. Intuitively, the transitions of $T_3'$ simulate the Petri net transitions of $T_3$, the transitions of $T_P$ guarantee that a terminal consensus is reachable from every configuration that does not represent $\mathbf {p_0}$, and the $T_s$ are additional silent actions to make the protocol well-formed.

The transitions of $T_3'$ simulate the behaviour of $N_3$. For this, $T_3'$ contains a transition $t'$ for every net transition $t \in T_3$. If $t \in T_3$ has two input places $p_1, p_2$ and two output places $p_1', p_2'$, then $t' = (p_1, p_2) \xrightarrow {} (p_1', p_2')$, The other cases are: if *t* has one input place $p_1$ and two output places $p_1', p_2'$, then $t' = (p_1, { Fresh}) \xrightarrow {} (p_1', p_2')$; if *t* has two input places $p_1, p_2$ and one output place $p_1'$, then $t' = (p_1, p_2) \xrightarrow {} (p_1', { Used})$; if *t* has one input place $p_1$ and one output place $p_1'$, then $t' = (p_1, { Fresh}) \xrightarrow {} (p_1', { Used})$.

The transitions of $T_P$ test the presence of tokens anywhere, apart from one single token in $p_0$. For every pair $(q, q') \in \left( \left( P_3 \setminus \left\{ p_0\right\} \right) \times Q \right) \cup \left\{ (p_0, p_0)\right\}$, the set $T_P$ contains a transition $(q, q') \mapsto ({ Collect}, { Collect})$. Further, for every place $q \in Q$, the set $T_P$ contains a transition $(q, { Collect}) \mapsto ({ Collect}, { Collect})$. Intuitively, these transitions guarantee that as long as the current marking of $N_3$ is different from $\mathbf {p_0}$, the protocol ${\mathcal {P}}$ can reach a terminal configuration with all agents in state ${ Collect}$.

The set $T_s$ contains a silent transition $(q, q') \mapsto (q, q')$ for every pair $(q, q')$ of states.

Assume that $\mathbf {p_0} \in { Reach}(N_3, M)$ for some marking *M* such that $M({\hat{p}}) = M(p_0) = M(P_{aux}) = 0$. Let $\sigma$ be a firing sequence such that $M \xrightarrow {\sigma } \mathbf {p_0}$. Observe that $\sigma$ is nonempty, and must end with a firing of transition $t_0$. Let *K* be the number of times that transitions with only one input place occur in $\sigma$. We claim that the initial configuration *C* given by $C({ Fresh})= K$, and $C(p)=M(p)$ for every $p \in P_3$ has a fair execution that does not reach a consensus. Indeed, the finite execution of ${\mathcal {P}}$ that simulates $\sigma$ by executing the corresponding transitions of $T_3'$ (and which, abusing language, we also denote $\sigma$), reaches a configuration $C'$ with $C'(p_0)=1$, $C'({ Fresh})=0$, $C'({ Used}) > 0$ (because every transition that moves an agent to $p_0$ also moves an agent to ${ Used}$), and $C'(p) = 0$ for any other place *p*. Since $O(p_0)= 1$ and $O({ Used})=0$, the configuration $C'$ is not a consensus configuration. Since no transition of $T_3' \cup T_P$ is enabled at $C'$, all transitions enabled at $C'$ are silent, and therefore from $C'$ it is not possible to reach a consensus.

Assume now that $\mathbf {p_0} \notin { Reach}(N_3, M)$ for any marking *M* such that $M({\hat{p}}) = M(p_0) = M(P_{aux}) = 0$. Then every configuration reachable from any initial configuration enables some transition of $T_P$. By fairness, every fair execution from any initial configuration contains at least one transition of $T_P$, and so some configuration reached along the execution populates state *Collect*. But then, again by fairness, the execution gets eventually trapped in a terminal configuration *C* of the form $C({ Collect})> 0$ and $C(q)=0$ for every $q \notin { Collect}$. So every fair execution is silent and stabilizes to 0, and therefore the protocol belongs to ${ WS}^2$. $\square$

### Proof that LayeredTermination is $\textsf {NP}$-hard

#### Proposition 21

LayeredTermination is $\textsf {NP}$-hard.

#### Proof

Let *X* be a finite set of variables and let $\textsf {C}$ be a set of 3-clauses defined over *X*, that is, the elements of $\textsf {C}$ are clauses of the form $(l_1 \vee l_2 \vee l_3)$ where $l_i \in X \cup \{\lnot x \mid x \in X\}$. The following problem (3-SAT) is known to be $\textsf {NP}$-complete: Given $\textsf {C}$, is $\bigwedge _{K \in \textsf {C}} K$ satisfiable?

We now show $\textsf {NP}$-hardness of LayeredTermination via a polynomial reduction from 3-SAT. To this end, we construct a protocol where agents can store either one of the following:

1. Variables $x \in X$ and their boolean assignments $x := b$ for $b \in \{0, 1\}$,
2. Clauses $K \in \textsf {C}$, along with variable assignments relevant to the satisfiability of the clause.

The assignments in the second case are collected from the other agents. If the collected assignment does not satisfy *K*, then assignments will eventually be reset, and agents may guess new assignments. If no satisfying assignment exists, then resets will occur infinitely often in some fair runs, which entails that LayeredTermination is not satisfied. Conversely, we will construct the protocol in a way that a decomposition into two layers satisfying LayeredTermination exists, whenever the 3-SAT instance is satisfied.

For a given literal *l*, let *X*(*l*) denote the variable contained in *l*. Case 1 above can be represented by states of the form *x* (*no assignment has been made*) and (*x*, *b*) (*assignment* $x := b$).

For every $K = (l_1 \vee l_2 \vee l_3)$ with $\ X(l_i) = x_i$ for $i \in \{1, 2, 3 \}$, we may represent Case 2 from above by states *K* (*no assignment collected*) and $(K, (b_1, b_2, b_3))$ (*assignment* $(x_1,x_2,x_3) := (b_1,b_2,b_3)$). Then we wish to add the transition

$$\begin{aligned} (x_1, b_1), (x_2, b_2), (x_3, b_3), K \mapsto (x_1, b_1), (x_2, b_2), (x_3, b_3), (K, (b_1, b_2, b_3)), \end{aligned}$$

if the assignment $(x_1,x_2,x_3) := (b_1, b_2, b_3)$ satisfies *K*, and otherwise

$$\begin{aligned} (x_1, b_1), (x_2, b_2), (x_3, b_3), K \mapsto x_1, x_2, x_3, K. \end{aligned}$$

However, this would require a 4-way interaction, instead of the usual pairwise ones, and so we have to emulate this behavior by a sequence of pairwise interactions that set/reset assignments in succession. To indicate the direction of the sequential movement (collect or reset), we replace *K* by $K_\rightarrow$ and $K_\leftarrow$, where $K_\rightarrow$ denotes the phase of collecting assignments, and $K_\leftarrow$ denotes that collected assignments should be successively reset.

For a given set of clauses $\textsf {C}$, we thus define a protocol with states

$$\begin{aligned} Q {\mathop {=}\limits ^{\text {def}}}\textsf {C}\cup X \cup (X \times \{0, 1\}) \cup \bigcup _{i\in \{1, 2, 3\}} \{K_{\leftarrow }, K_{\rightarrow } \mid K \in \textsf {C}\} \times \{0, 1\}^i \end{aligned}$$

and transitions

$$\begin{aligned} T = T_{\mathtt {assign}} \cup T_{\mathtt {reset}} \cup T_{\mathtt {clause}}. \end{aligned}$$

We will now define the transitions. To establish LayeredTermination, neither the input nor the output is required. We thus omit the specification of the input and output of the protocol; for the purpose of this proof, they can be given arbitrarily.

The transitions from $T_{\mathtt {assign}}$ assign a boolean value to every $x \in X$. The set $T_{\mathtt {assign}}$ is constructed as follows. For every $x \in X$, $b \in \{0, 1\}$ and $q \in Q$, we add the following transition to $T_{\mathtt {assign}}$:

$$\begin{aligned} (x, q) \mapsto \left( (x, b), q\right) \end{aligned}$$

The transitions from $T_{\mathtt {clause}}$ collect the assignments that are relevant to each clause, and trigger a reset when the assignment is not satisfied. The set $T_{\mathtt {clause}}$ is constructed as follows. For every clause $(l_1 \vee l_2 \vee l_3) \in \textsf {C}$ with $\mathbf {x} = (x_1, x_2, x_3) = (X(l_1), X(l_2), X(l_3))$, and every $(b_1, b_2, b_3) = \mathbf {b} \in \{0, 1\}^3$, we add the following transitions:

$$\begin{aligned} \left( K, (x_1, b_1)\right)&\mapsto \left( (K_\rightarrow , b_1), (x_1, b_1)\right) \\ \left( (K_\rightarrow , b_1), (x_2, b_2)\right)&\mapsto \left( (K_\rightarrow , (b_1, b_2)), (x_2, b_2)\right) \\ \left( (K_\rightarrow , (b_1, b_2)), (x_3, b_3)\right)&\mapsto {\left\{ \begin{array}{ll} \left( (K_\rightarrow , \mathbf {b}), (x_3, b_3)\right) &{} \text { if assignment } \mathbf {x} := \mathbf {b} \text { satisfies } K, \\ \left( (K_\leftarrow , \mathbf {b}), (x_3, b_3)\right) &{} \text { otherwise.} \end{array}\right. } \end{aligned}$$

Finally, the transitions from $T_{\mathtt {reset}}$ reset assignments that do not satisfy a given clause. For every $(l_1 \vee l_2 \vee l_3) = K \in \textsf {C}$ where $x_i = X(l_i)$, and every $(b_1, b_2, b_3) = \mathbf {b} \in \{0, 1\}^3$, we add the following transitions to $T_{\mathtt {reset}}$:

$$\begin{aligned} \left( (K_\leftarrow , \mathbf {b}), (x_3, b_3)\right)&\mapsto \left( (K_\leftarrow , (b_1, b_2)), x_3\right) \\ \left( (K_\leftarrow , (b_1, b_2)), (x_2, b_2)\right)&\mapsto \left( (K_\leftarrow , b_1), x_2\right) \\ \left( (K_\leftarrow , b_1), (x_1, b_1)\right)&\mapsto \left( K, x_1\right) \end{aligned}$$

Moreover, for every $\circ \in \{\leftarrow , \rightarrow \}$, $1 \le i \le j \le 3$, $b \in \{0, 1\}$, $\mathbf {b} \in \{0, 1\}^j$, and $K = (l_1 \vee l_2 \vee l_3) \in \textsf {C}$, $x = X(l_i)$, such that $b \ne b_i$, we add this transition to $T_\mathtt {reset}$:

$$\begin{aligned} \left( (K_\circ , \mathbf {b}), (x, b)\right) \mapsto (K, x). \end{aligned}$$

Assume there exists a satisfying assignment $\sigma :X \xrightarrow {} \{0, 1\}$ for $\bigwedge _{K \in \textsf {C}} K$ . Then it is easy to verify that the following partition $T = T_1 \cup T_2$ proves LayeredTermination:

$$\begin{aligned} T_1&{\mathop {=}\limits ^{\text {def}}}\{x, q \xrightarrow {} (x, \sigma (x)), q \mid x \in X, q \in Q\} \cup T_{\mathtt {reset}} \cup T_{\mathtt {clause}}\\ T_2&{\mathop {=}\limits ^{\text {def}}}T_{\mathtt {assign}} \setminus T_1 \end{aligned}$$

We have to show that every execution of ${\mathcal {P}}[T_1]$ and ${\mathcal {P}}[T_2]$ is silent, and that transitions from $T_2$ cannot reenable $T_1$. Clearly, transitions from $T_2$ cannot reenable $T_1$, and every execution of ${\mathcal {P}}[T_2]$ is clearly silent. It remains to show that every execution of ${\mathcal {P}}[T_1]$ is silent. By inspection of *T*, we have that every non-silent execution must contain infinitely many transitions from both $T_\mathtt {reset}$ and $T_{\mathtt {assign}}$. But $T_1$ only contains those transitions from $T_{\mathtt {assign}}$ that agree with $\sigma$. Thus, every time an assignment is reset and a variable is reassigned, the number of agents disagreeing with $\sigma$ is reduced. Eventually the transitions from $T_\mathtt {collect}$ only pick up assignments agreeing with $\sigma$. Since $\sigma$ is a satisfying assignment, all transitions from $T_\mathtt {reset}$ are eventually disabled forever. However, recall that the number of occurrences of transitions from $T_\mathtt {reset}$ is infinite in non-silent executions, and so we conclude that all executions of ${\mathcal {P}}[T_1]$ are silent. This shows that LayeredTermination is satisfied whenever some satisfying assignment $\sigma$ exists.

Conversely, if there is no satisfying assignment, then LayeredTermination is not satisfied: In this case, it is easy to verify that every fair execution starting in a configuration *C*, such that $\llbracket C\rrbracket = X \cup \textsf {C}$ and $C(q) = 1$ for every $q \in \llbracket C\rrbracket$, must be non-silent, hence LayeredTermination must be violated. $\square$

## Footnotes

1 While protocols only model interactions between two agents, *k*-way interactions for a fixed $k > 2$ can be simulated by adding additional states [8].

2 Silent protocols are also referred to as *protocols with stabilizing states* and silent transitions are called *ineffective* in [26, 27].

3 In a logical context these mappings are called valuations instead of populations.

4 [13] gives an equivalent formula with threshold and divisibility constraints, but the divisibility constraints can be easily replaced by remainder constraints.

5 Peregrine and benchmarks are available from https://gitlab.lrz.de/i7/peregrine/.

6 The variant from [12] is referred to as *threshold-n* by its authors.
